# In no uncertain terms: Group cohesion did not affect exploration and group decision making under low uncertainty

**DOI:** 10.3389/fpsyg.2023.1038262

**Published:** 2023-01-25

**Authors:** Marie Ritter, Johannes Pritz, Lara Morscheck, Emma Baumann, Margarete Boos

**Affiliations:** Social and Communication Psychology, Georg-Elias-Müller-Institute for Psychology, University of Göttingen, Göttingen, Germany

**Keywords:** group cohesion, exploration, group decision making, uncertainty, HoneyComb paradigm, leadership, collective induction, ε-greedy

## Abstract

Group decision making under uncertainty often requires groups to balance exploration of their environment with exploitation of the seemingly best option. In order to succeed at this collective induction, groups need to merge the knowledge of all group members and combine goal-oriented and social motivations (i.e., group cohesion). This paper presents three studies that investigate whether more cohesive groups perform worse at collective induction tasks as they spend less time exploring possible options. Study 1 simulates group decision making with the ε-greedy algorithm in order to identify suitable manipulations of group cohesion and investigate how differing exploration lengths can affect outcomes of group decisions. Study 2 (*N* = 108, 18 groups á 6 participants) used an experimental manipulation of group cohesion in a simple card choice task to investigate how group cohesion might affect group decision making when only limited social information is available. Study 3 (*N* = 96, 16 groups á 6 participants) experimentally manipulated group cohesion and used the HoneyComb paradigm, a movement-based group experiment platform, to investigate which group processes would emerge during decision making and how these processes would affect the relationships between group cohesion, exploration length, and group decision making. Study 1 found that multiplicative cohesion rewards have detrimental effects on group decision making, while additive group rewards could ameliorate negative effects of the cohesion reward, especially when reported separately from task rewards. Additionally, exploration length was found to profoundly affect decision quality. Studies 2 and 3 showed that groups could identify the best reward option successfully, regardless of group cohesion manipulation. This effect is interpreted as a ceiling effect as the decision task was likely too easy to solve. Study 3 identified that spatial group cohesion on the playing field correlated with self-reported entitativity and leader-/followership emerged spontaneously in most groups and correlated with self-reported perceptions of leader-/followership in the game. We discuss advantages of simulation studies, possible adaptations to the ε-greedy algorithm, and methodological aspects of measuring behavioral group cohesion and leadership to inform empirical studies investigating group decision making under uncertainty.

## 1. Introduction

From an evolutionary perspective, humans naturally live in groups in which individual members contribute to group tasks (van Vugt and Ronay, 2014). This creates an advantage as individuals can share resources, labor, and knowledge (Kozlowski and Chao, 2012) and less experienced group members can learn successful behavior from others, avoiding the costs of trial-and-error (Rendell et al., 2010; Mesoudi, 2011; Wisdom et al., 2013). When groups make decisions in an uncertain environment, they can draw on the knowledge or experience of individual members in order to learn about their decision options. This has been termed collective cognition (Couzin, 2009) or collective induction (Laughlin and Hollingshead, 1995; Laughlin, 1999). Understanding how collective induction emerges remains one of the central questions of unraveling group decision making under uncertainty (King and Sueur, 2011; Grand et al., 2016) and phenomena like groupthink (Janis, 2008) suggest that group cohesion might play an important role. The aim of this paper is to investigate the role of group cohesion in group decision making under uncertainty and illuminate the emergent mechanisms behind it. Specifically, we use three studies to examine whether more cohesive groups perform worse as they forego chances of exploring different options in an uncertain environment.

While the relationship between group cohesion and performance has been researched extensively over the last years, findings are all but consistent (Casey-Campbell and Martens, 2009). Some studies suggest that high group cohesion will improve group communication (i.e., information transfer), lead to effort gains, and higher performance (Cartwright, 1968). It has been shown that groups who successfully pool private information, so information that is available to individual members, achieve higher performance or efficiency in decision making (Tomasello, 1999; Helbing et al., 2000; Couzin et al., 2002; Boyd et al., 2011; King and Sueur, 2011; Laughlin, 2011; Van Vugt and Kameda, 2012; Moussaïd et al., 2016; Sridhar et al., 2021). Further, tightly knit social networks facilitate the exchange of information and enhance group performance (Mason and Watts, 2012; Derex and Boyd, 2015). In moving groups, group cohesion (i.e., staying closely together) can facilitate the “pooling […] from many inaccurate compasses” (Simons, 2004) and help less knowledgeable individuals prioritize social information (i.e., information that is shared by other group members) and motivations above their own private information and goal-directed behavior (Boos et al., 2014; Sridhar et al., 2021). Individuals might balance social and goal-oriented motivations by observing their neighbors and adapting their own movement direction accordingly (Couzin and Krause, 2003; Conradt and Roper, 2009; Sridhar et al., 2021).

In contrast, it has been shown that group cohesion can negatively impact group decision performance and information transfer (March, 1991; van Ginkel and van Knippenberg, 2012; Mehlhorn et al., 2015). Some studies suggest that sparser or loosely coupled social networks sometimes outperform more connected ones (Mason et al., 2008; Fang et al., 2010; Derex and Boyd, 2016). The detrimental effect of group cohesion is most pronounced when decision speed is prioritized (Gavrilets and Richerson, 2017; Yahosseini et al., 2018), as can be seen in phenomena like groupthink (Janis, 1972, 2008) or hidden-profile experiments (Stasser and Titus, 2003). Recent research suggests that the context of a group or team likely determines whether group cohesion positively or negatively affects effort (Torka et al., 2021) or group decision making (Casey-Campbell and Martens, 2009). Perhaps paradoxically, it has been shown that individuals are more motivated to follow a group when they experience uncertainty (Hogg, 2000), aggravating the already disadvantageous effect of group cohesion.

In sum, when social orientation (group cohesion) outweighs goal orientation, group members might forego their personal preference to stick with the group (e.g., Sridhar et al., 2021). This means that cohesive groups might miss important information. On the other hand, however, completely disjoint groups will not be able to share information between group members to engage in collective induction (Laughlin, 1999) and to build collective information. This means that the “right balance of interdependence and independence” (Conradt, 2012, p. 1) appears crucial.

A key element in the relationship between group cohesion and group decision performance is the amount of exploration undertaken by the group or its individual members. The decision to stop accumulating more information about the environment (exploration) and commit to one option (exploitation) is a central challenge for individuals (Cohen et al., 2007; Tickle et al., 2021). Individuals faced with an uncertain decision task will usually engage in exploration in the beginning before transitioning to exploitation of the option they estimate to be best (Bechara et al., 1994). This has been investigated in computational models of reinforcement learning (e.g., ε-greedy algorithms; Sutton and Barto, 2018) that predict the best outcomes for a medium amount of exploration (i.e., an inverted U-relationship between exploration and decision outcome). However, if group cohesion is high, individual group members might rely on the knowledge of others instead of exploring on their own (Bolton and Harris, 1999; Yahosseini et al., 2018). It has been shown that high group cohesion and resulting mutual reinforcement of sub-optimal choices prevents groups from exploring more profitable options (Bala and Goyal, 1998; Giraldeau et al., 2002; Salganik and Watts, 2008).

Ritter et al. (2021) used the HoneyComb paradigm, a multi-agent virtual game platform, to investigate conditions under which humans are able to identify the most advantageous leader. In the experiment, the leaders were four pre-programmed agents and differed in expected values (i.e., in the probability and amount of pay-out). In order to infer which leader was best, participants had to explore by following the leaders in repeated interactions (i.e., 30 game rounds). After an initial exploration period, participants settled for one leader and exploited this option for the remaining part of the game. Within this experiment, three experimental conditions were tested: In the single condition, one participant played alone. In the independence condition, six participants played the game at the same time and each participant could simultaneously observe the movement of all other players. In the cohesion condition, six participants played the game as in the independence condition but received a cohesion reward for following a leader together with other participants. The incentive was implemented by multiplying the reward gained from the leader with the number of participants who had followed this leader. It was found that participants in the cohesion condition were more likely to settle on a less advantageous leader (Ritter et al., 2021). While the effect of the implemented reward system (multiplicative cohesion reward) drove this effect, exploratory results suggested that high group cohesion might negatively affect group decision making.

In the current paper, we want to investigate whether increased group cohesion has a detrimental effect on group decision making by prioritizing social information over individual exploration, as previous work suggests (e.g., Yahosseini et al., 2018). To this end, we conducted three studies with increasing complexity: In the first study, we aimed to identify key parameters of the group decision making process under uncertainty using a reductionist approach in a simulation study. We use the ε-greedy algorithm (Sutton and Barto, 2018) to substantiate claims about the detrimental effect of the multiplicative cohesion reward structure on group decision making (Ritter et al., 2021) and propose an adjusted cohesion reward structure. Additionally, we demonstrate the effects of exploration on group decision making to inform the two following behavioral studies. Based on findings of Study 1, we designed the behavioral group decision making experiment tasks used in Study 2 and 3.

In the second study, we empirically investigate in a reductionist behavioral experiment whether groups who are incentivized to behave cohesively perform worse at a collective induction task, and whether this can be attributed to differences in exploration times. To test this prediction, we used a repeated card-choice task (similar to the Iowa Gambling Task, Bechara et al., 1994). This reductionist experiment design allows us to transfer findings of the simulation in Study 1 back to human behavior while keeping complexity of the task at a minimum. Participants explored the options individually but were given social information (i.e., information about the choices of other group members). The study investigated whether participants in groups incentivized to choose the same card would prioritize social information over individual exploration.

In the third study, we investigate in a movement-based behavioral experiment the emergent group processes that affect group decision making. We do so by implementing the reductionist decision task of Study 2 within the HoneyComb paradigm (Boos et al., 2019) that was previously used by Ritter et al. (2021). In this study, players were faced with the same choice options as in Study 2 but could communicate through movement on the playing field. All other verbal and non-verbal communication was blocked. The added possibility to communicate increases the complexity compared to Study 2. In this way, Study 3 builds on all previous results (Ritter et al., 2021; as well as Study 1 and Study 2). By using the HoneyComb paradigm, we were able record spatio-temporal data to observe the group processes in real time. With these three studies, increasing in complexity, we aim to answer the basic question: How does group cohesion affect group decision making under uncertainty? In Study 1, we provide a detailed view of group decision making processes dependent on key parameters, such as exploration length, through a simulation study. In Study 2, we investigate this question in a simplistic choice task to investigate the influence of basic social information. In Study 3, we extend the choice task of Study 2 to a movement paradigm in order to investigate in detail which emerging group processes can be identified in group decision making under uncertainty. In this way, these three studies address the question at hand with increasing complexity.

## 2. Study 1

The purpose of this study was to identify key parameters of the group decision making process as investigated by Ritter et al. (2021). They implemented a multiplicative cohesion reward: In the cohesion condition, the rewards were multiplied by the number of participants arriving at the same reward field. Due to this reward inflation, participants in the cohesion condition were not able to accurately infer the value of the different options. We aim to substantiate these claims using a reductionist simulation approach and identify additional parameters that could affect collective induction. To this end, we adapted the ε-greedy algorithm, a popular reinforcement learning algorithm, to model decision making under uncertainty (Sutton and Barto, 2018). The ε-greedy algorithm serves as a formalization of the previously used group decision making task (Ritter et al., 2021) in which exploration/exploitation trade-offs had to be made by a group. As a detailed account of the ε-greedy algorithm is beyond the scope of this paper, we refer interested readers to introductory literature (e.g., Sutton and Barto, 2018) and limit ourselves to a basic explanation of the simulation algorithm, its parameters, and its relation to our psychological research question.

The ε-greedy algorithm is a reinforcement learning optimization method. It is often applied to so-called multi-armed bandit problems that are used to investigate decision making under uncertainty. A multi-armed bandit problem contains *k* different options and one agent that aims to find the best option. All *k* options have underlying reward distributions that are unknown to the agent. The multi-armed bandit problem can be extended to include multiple agents *n*: a multi-agent multi-armed bandit problem. In order to maximize their reward, agents need to discover the option that yields the highest reward through iterated trial-and-error choices (exploration). An important assumption of the basic ε-greedy algorithm is that the reward distributions underlying each of the options are stationary (i.e., are not subject to change from one iteration to the next). This means that each option follows a predefined reward distribution and that the parameters defining these distributions should not change. For example, the option of following the competent leader (Ritter et al., 2021) was defined by a binomial distribution with 80% success rate (*C*∼ *B(n, p)*, *p* = 0.8, *n* = 30 rounds). The expected value of this option *E[C]*, in the independence condition, was *E[C]* = n *p = 24 multiplied by the amount of payout E[C] *20 cent = 480 cent. To satisfy the assumption of stationary reward distributions, this distribution should be constant across all iterations (or rounds). In principle, the Ritter et al.’s (2021) experiment task constitutes a multi-armed bandit problem: The four leaders are the different options (*k* = 4) and their underlying reward probability is unknown to participants at the beginning of the game. The six players are the agents (*n* = 6) and they need to repeatedly choose from the four options in order to infer the option that yields the highest reward. Importantly, the assumption of stationary reward distributions is met in the independence condition (i.e., no cohesion reward), but not in the cohesion condition (i.e., multiplication of rewards with number of participants choosing the same option). In the cohesion condition, the presence of others following the same leader affected the reward that could be gained from an option. This could be one reason for the suboptimal performance of participants in the cohesion condition. In the cohesion condition, participants’ rewards incorporated pay-out from the leaders, on the one hand, and the cohesion reward, on the other hand, without separating the feedback from these two sources. This means that agents in the cohesion condition received information that was corrupted by the cohesion reward and was, therefore, not suited to reliably estimate the underlying reward distributions.

In a multi-armed bandit problem, the ε-greedy algorithm is one possible way to find the best out of the *k* options. The *n* agents strive to maximize their (numerical) reward gained from different choices. To do so, they need to balance exploring the different options and exploiting the option that seems best in a given iteration. This is implemented in the following way: Each agent starts out by assuming that all *k* options are equally profitable. With each iteration (or each choice) an agent makes, their knowledge about the different options is updated and will become increasingly accurate. We can find analogous processes in human decision making under uncertainty. For example, the updating of information one holds about different options enables human decision makers to infer the best card stack in the Iowa Gambling Task (Bechara et al., 1994). Additionally, it has been shown that neural networks in the human brain encode accumulated evidence about different options in the form of estimated distributions (Beck et al., 2008; Churchland et al., 2008).

In order to refine their knowledge, agents need to explore different options (i.e., make a random choice independent of previous experience). The tendency to explore is implemented with the ε-parameter (0 < ε < 1). For each iteration *t* of the algorithm (i.e., every time an agent needs to make a choice), a random number *p* is drawn from a uniform distribution between 0 and 1. If *p* < ε, the agent will randomly choose one of the options; if *p* > ε, the agent will choose the option with the highest expected reward based on their current knowledge. This means that a small ε will result in few exploratory iterations (e.g., ε = 0.01: about 1% of trials will be used for exploration), a large ε in many exploratory iterations (e.g., ε = 0.5: about 50%). Due to the random nature of determining whether an iteration will be used for exploration or exploitation, exploration can also happen at late stages of the algorithm, contrary to findings about human exploration/exploitation patterns (Bechara et al., 1994). Nonetheless, we argue that this algorithm is well suited to model known psychological repeated choice problems. In this way, the ε-greedy algorithm can provide an interesting point of comparison to decision making problems under uncertainty.

### 2.1. Simulation

Starting from an existing Python script (Samishawl, 2020), we set up an ε-greedy simulation to include six agent (*n* = 6) and four options (*k* = 4). The four options followed the same reward distributions as in Ritter et al.’s (2021) study as can be seen in Table 1. Additionally, three different bonus types were implemented, corresponding to the different conditions (Ritter et al., 2021): (a) no group bonus, (b) multiplicative group bonus (cohesion condition, reward multiplied by number of agents choosing an option), and (c) a new additive group bonus. With the additive group bonus, agents receive a fixed bonus (3 cent) for each other agent that chooses the same option. With the additive bonus, rewards are less inflated, compared to the multiplicative bonus. We included the additive bonus to explore whether it could be a viable alternative to the multiplicative cohesion reward in future experiments. Simulations included 30 iterations (*t_*max*_* = 30), corresponding to the 30 rounds in the previous study. To compare the three reward structures, ε was kept constant (ε = 0.1) across three simulations. Additionally, we explored the influence of the ε parameter by running five different simulations (ε ∈ {0.01, 0.05, 0.1, 0.2, 0.5}). For these comparisons, we did not include any group reward to disentangle the effects of reward structure and exploration rates. In all simulations, agents explored for the first 3 iterations before continuing with the algorithm as described above. We recorded the number of agents choosing each option for each iteration of each simulation. Each simulation was repeated for 1,000 runs (corresponding to groups in a human experiment). The Python script used for simulations, resulting data, and the analysis script can be found on our OSF project.^1^ Please note that all included parameters (number of agents, number of options, distributions, number of rounds) were chosen to model the experimental conditions of the study by Ritter et al. (2021).

**TABLE 1:** Overview over experimental conditions and options in Study 1–3.

	Ritter et al. (2021)/ Study 1	Study 2	Study 3
Task	Movement paradigm (HoneyComb)/ Simulation	Card-choice task	Movement paradigm (HoneyComb)
Response format	Follow pre-programmed leader	Choose card stack	Move to reward field
Social information	Movement Local visual radius	Minimal Feedback of others’ choices	Movement Global visual radius
Conditions	Single, independence, cohesion	Independence, cohesion	Independence, cohesion
Cohesion reward	Multiplicative	Additive (3 cent)	Additive (3 cent)
Options	Option name Payout; Probability Expected value		
Most profitable	Profitable leader 20 cent; 80% 480 cent	Profitable stack 30 cent; 80% 720 cent	Profitable field 30 cent; 80% 720 cent
Secure neutral	Secure neutral leader 10 cent; 90% 270 cent	Secure neutral stack 10 cent; 90% 270 cent	Secure neutral field 10 cent; 90% 270 cent
Risky neutral	Risky neutral leader 20 cent; 45% 270 cent	Risky neutral stack 30 cent; 30% 270 cent	Risky neutral field 30 cent; 30% 270 cent
Least profitable	Unprofitable leader 20 cent; 20% 120 cent	Unprofitable stack 10 cent; 20% 60 cent	Unprofitable field 10 cent; 20% 60 cent

### 2.2. Results

**FIGURE 1:**
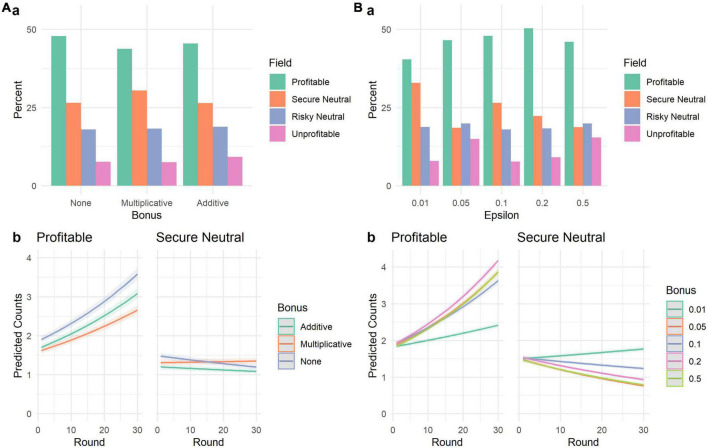
Results of Study 1. (**A**–upper panel) shows how often each field was chosen (in percent) by 6 simulated agents (30 rounds, 1,000 runs) with no group bonus (“None”), an additive bonus, and a multiplicative bonus. The lower panel **(A–b)** shows the predicted number of agents choosing the profitable or secure neutral field, depending on round and group bonus. (**B**–upper panel) shows how often each field was chosen (in percent) by 6 simulated agents (30 rounds, 1,000 runs) with different exploration parameters (Epsilon). The lower panel **(B–b)** shows the predicted number of agents choosing the profitable or secure neutral field, depending on round and exploration length.

Results can be seen in Figure 1. We fitted a Poisson mixed model (estimated using ML and BOBYQA optimizer) to predict the number of agents choosing the profitable field with bonus type (none vs. multiplicative vs. additive) and iteration, with simulation run as random effect. There was a significant effect of round, indicating that agents learned to choose the profitable option (β = 0.02, 95% CI [0.02, 0.02], *p* < 0.001; std. β = 0.18, 95% CI [0.17, 0.18]). Interaction effects showed that agents identified the best option faster when receiving no bonus (β = 0.001, 95% CI [0.00, 0.002], *p* = 0.019, std. β = 0.01), compared to the multiplicative and additive bonus, and slower when receiving a multiplicative bonus (β = −0.003, 95% CI [−0.004, −0.002], *p* < 0.001, std. β = −0.03), compared to the no bonus and additive bonus runs. For the secure neutral option, the main effect of round reversed (β = −0.003, 95% CI [−0.004, −0.002], *p* < 0.001; std. β = −0.03, 95% CI [−0.04, −0.02]) as well as the interaction effects; multiplicative bonus: β = 0.004, 95% CI [0.003, 0.005], *p* < 0.001; std. β = 0.04, 95% CI [0.03, 0.05]; no bonus: β = −0.003, 95% CI [−0.005, −0.002], *p* < 0.001; std. β = −0.03, 95% CI [−0.05, −0.02]. More details on the regression analysis can be found in the Section “1.1.1. Study 1” in Supplementary material.

While the overall differences between the frequency of chosen options seem very small, the regression analyses replicate the previous general finding (Ritter et al., 2021): Runs that included the multiplicative bonus performed worse, compared to runs with no such reward. The additive bonus, while still producing suboptimal decisions, performs better, compared to the multiplicative reward. It is important to keep in mind that these results are produced by an algorithm designed to optimize rather than simulate decision problems, using the same decision task parameters as in the experimental study. Yet, the resulting patterns are comparable to those that can be found within human behavior (Ritter et al., 2021). Please note that no sensitivity analysis regarding the chosen parameters (e.g., number of agents, number of options) as the main aim of this study was to model the experimental conditions of the study by Ritter et al. (2021).

Lastly, we explored how different exploration rates influence the decision making process. Simulations using intermediate levels of exploration usually fared best in (a) terms of overall choices and (b) learning rates in choosing the profitable and secure neutral options. These observations are corroborated by quantitative analyses (see Section “1.1.1. Study 1” in Supplementary material) that consistently show no main effects for different ε-levels but interactions with round. This was true for both the number of agents choosing the profitable and the secure neutral option.

Results of this simulation study showed that simulated groups receiving a multiplicative or additive group bonus performed worse compared to groups receiving no such reward. However, when rewarding cohesion is a necessary experimental manipulation, an additive bonus should be implemented for better performance. Additionally, we advise that the bonus is reported separately from the choice reward (i.e., the reward stemming from the choice itself). Lastly, this study showed that the length of exploration is an important key element of group decision making under uncertainty that should be investigated in human behavior. This was done in Study 2.

## 3. Study 2

The aim of Study 2 was to investigate (a) whether more cohesive groups would perform worse in a group decision task, as found in previous work (Ritter et al., 2021), and (b) whether this effect can be attributed to differences in exploration (Yahosseini et al., 2018). In order to disentangle the effects of collective induction (Laughlin, 1999, 2011) and group cohesion, we amended earlier methodological limitations of the study by Ritter et al. (2021) by using the results of Study 1 to create a suitable reward structure. This study aims to transfer findings of Study 1 back to the study of human behavior while keeping the complexity of the experiment to a minimum. In the current study, the feedback about how much money participants earned in a given round is shown separately for earnings from the reward field and the cohesion incentive. By separating the feedback, the reward distributions are constant across all rounds, eliminating problems of information occlusion (non-stationary reward distributions) as discussed in Study 1. The experimental design in the current study is restricted to two conditions: the independence and cohesion condition. Cohesion was manipulated using an additive cohesion reward (i.e., 3 cents for each additional player on the same reward field). It should be noted that groups in the independence condition might also exhibit some group cohesion, albeit less compared to groups in the cohesion condition. This should be ensured using a manipulation check. Additionally, we transformed the original task (Ritter et al., 2021) into a card-choice paradigm (similar to the Iowa Gambling Task; Bechara et al., 1994): The differently profitable leaders were transformed into differently profitable card stacks that participants chose from. In this way, this study excludes more complex emergent processes (e.g., leadership) to focus on clearly discernible effects of cohesion on the use of social information. More complex group processes are investigated in Study 3. A comparison of design aspects of the study by Ritter et al. (2021), Study 2, and Study 3 can be seen in Table 1.

In Study 2, the group decision process is investigated under minimal interaction. In the previous experiment (Ritter et al., 2021), participants were able to communicate their decision preferences through movement, and possibly, use leader-/followership processes to guide group decision making. In contrast, communication between participants is blocked entirely in the current study. Participants were only informed about the choices other group members made. Therefore, the only social information available to participants was feedback about their own and other group members’ choices.

This investigation aimed to replicate findings indicating a detrimental effect of group cohesion on group decision making (H1–4; March, 1991; van Ginkel and van Knippenberg, 2012; Mehlhorn et al., 2015; Ritter et al., 2021) and the mediating role of exploration (H5–6; e.g., Yahosseini et al., 2018) as discussed in the theoretical background. The following hypotheses were formulated:

*H1*: Subjects will be more likely to find the profitable stack with increasing number of game rounds.

*H2*: There will be a stronger increase in finding the profitable stack with increasing number of rounds in the independence condition, compared to the cohesion condition.

*H3*: Subjects in the cohesion condition will have a shorter exploration phase measured by the half-change round (Ritter et al., 2021), compared to those in the independence condition.

*H4.1*: Subjects will choose worse options overall, measured by choice score (Ritter et al., 2021) in the cohesion condition, compared to those in the independence condition.

*H4.2*: Overall, subjects in the cohesion condition will choose the profitable stack less often, but the safe neutral stack more often compared to those in the independence condition.

*H5*: Participants with a longer exploration phase determined by the half-change round will make better decisions overall. We anticipate an inverted U-shape as predicted by ε-greedy algorithms (e.g., Sutton and Barto, 2018). The inverted U-shape suggests that the highest quality of decision occurs at an intermediate level of exploration while low and high levels of exploration will result in impaired decision quality. We therefore expect a negative square correlation between exploration and decision quality.

*H6.1*: The relationship between condition (cohesion vs. independence) and the quality of decision making is mediated by the length of the exploration phase.

*H6.2*: The relationship between condition (cohesion vs. independence) and choice of the profitable stack is mediated by the length of the exploration phase.

### 3.1. Methods

This study is designed as a mixed-design with a 2-level between-subjects manipulation (independence vs. cohesion condition) with repeated measures (30 rounds).

#### 3.1.1. Sample

In this study, 108 participants (84 women, 23 men, 1 diverse; Age: *M* = 22.96 years, *SD* = 5.11) played the game in groups of six, resulting in a total of 18 groups (power analyses in Section “1.2. Power Simulation” in Supplementary material; additional sample information see Section “1.3.1. Study 2” in Supplementary material). All data collection procedures were approved by the Ethics Committee of the Georg-Elias-Müller-Institute for Psychology (proposal 305/2022).

#### 3.1.2. Procedure

Once participants signed up for the study, they received an e-mail with a link to an online meeting room (BigBlueButton). At the start of the experiment time slot, participants were asked to join this online meeting. To preserve anonymity, participants were prohibited from sharing their camera, microphone, or name. This meeting served to ensure that all six participants were present and to create social presence within the participant group. After some initial instructions, participants received a link to a form in which they had to give written consent in order to participate. Subsequently, another link was sent to participants that led them to the online experiment programmed within Labvanced (Finger et al., 2017).

**FIGURE 2:**
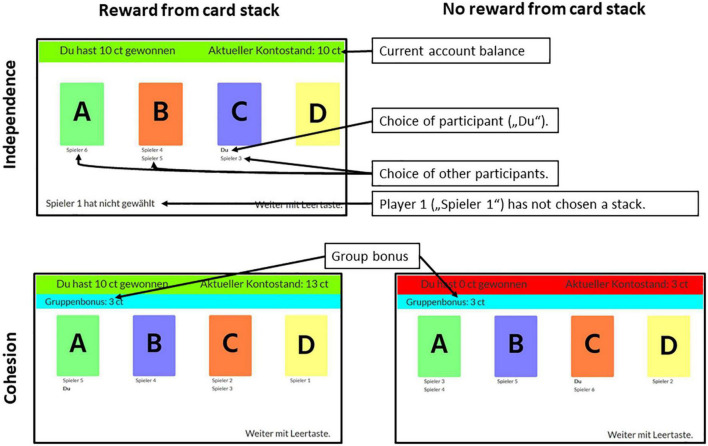
Screenshots from the experiment as seen by participants. The **(upper)** screenshot shows a screen in the independent condition when the participant received a pay-out for choosing a card (green bar). The **(lower)** screenshots show the cohesion condition in which participants received a group bonus (blue bar) and received payout (**left**, green bar) or did not receive payout (**right**, red bar). Card stacks were colored differently and colors were randomly assigned in the beginning of each game. Participants were naïve to the exact pay-out probabilities and amounts and had to infer the best stack through exploring the different card stacks.

Participants were shown instructions that explained that they would play a game during which no communication with the other participants would be allowed or necessary. It was explained that the game consists of repeated card choices with each card choice representing one round (see Figure 2).

The task of the participants was to earn as much money as possible by choosing cards from four card stacks labeled “A,” “B,” “C,” and “D.” The general setting consisted of four different stacks with different expected values (Table 1 and Figure 2). In each round, participants had to choose from which card stack they wanted to draw with a mouse-click. A colored bar (green for pay-out, red for no pay-out) at the top of the screen informed them whether the card had paid out and, if so, how much. If participants failed to choose a card in the allotted time (40 s), the red bar showed the message “YOU DID NOT CHOOSE.” Participants who had not chosen a card in a given round did not receive payout in this round. Additionally, the feedback screen displayed the choices of all other players by printing the player number under the card they had chosen. This was done to provide a minimum of social information to participants. The player’s own choice was shown by printing “You” under the chosen card. In the cohesion condition, participants received an additional bonus of 3 cents for each other participant that had chosen the same card. A blue bar at the top of the screen informed participants about the earned group bonus so that the reward from the card choice and the group bonus were presented separately. In the independence condition, no such bonus was implemented. As in the study by Ritter et al. (2021), the participants played 30 repeated rounds of this game. After the last round, participants were informed about the total amount of money they had earned and were then led to a post-experiment questionnaire. Participants were then asked to return to the online meeting room, where they were thanked and dismissed.

#### 3.1.3. Operationalization

***Decision quality*** is operationalized in two ways: choice of the profitable stack in each round and an overall choice score to quantify overall decision quality in the game. The *choice of profitable stack* is a binary variable in each round and the probability to choose the profitable stack (i.e., card stack with highest expected value) will be calculated within a logistic regression model. The *choice score* is the overall quality of card stack choices which is determined by a cumulative points system (Ritter et al., 2021). To calculate the choice score, points were assigned to each card choice and summed over all rounds: For the profitable stack, participants received 3 points, for the neutral stacks, participants received 2 points, for the unprofitable stack, participants received 1 point. If participants failed to choose a card stack, they received 0 points. Note that the choice score is simply an overall operationalization of participants’ decision quality for analysis purposes and was not used to calculate participants’ earnings.

***Group cohesion*** is operationalized as the manipulated independent variable condition. Half of the groups were incentivized with a group bonus to show group cohesion (cohesion condition), while the other half was not incentivized in this way (independence condition).

***Exploration*** is operationalized using the *half-change-round* (Ritter et al., 2021): the round in which at least half of all card stack changes of one participant had occurred. It is used to operationalize the end of the exploration phase and beginning of the exploitation phase.

#### 3.1.4. Data preprocessing and analysis

Data was preprocessed and analyzed using R, running in RStudio (R Core Team, 2020; RStudio Team, 2020). Used R packages can be found in the Section “1.4. R packages” in Supplementary material. The analysis script and preprocessed data can be found on the OSF project (see text footnote 1).

### 3.2. Results

**H1**. As expected, participants were indeed significantly more likely to choose the profitable card stack in later rounds, compared to earlier rounds (β = 0.04, 95% CI [0.02, 0.06], *p* < 0.001; std. β = 0.62, 95% CI [0.39, 0.86]). To check this, we fitted a logistic mixed model (estimated using ML and BOBYQA optimizer) to predict choice of the profitable card stack with round (formula: choice of profitable card stack ∼ −1 + round). The model included round, participant id and group as random effects. The model’s total explanatory power is substantial (conditional *R*^2^ = 0.39) and the part related to the fixed effects alone (marginal *R*^2^) is of 0.02. Standardized parameters were obtained by fitting the model on a standardized version of the dataset. 95% Confidence Intervals (CIs) and *p*-values were computed using a Wald z-distribution approximation. All models reported in the following are fitted in the same way if not stated otherwise.

**FIGURE 3:**
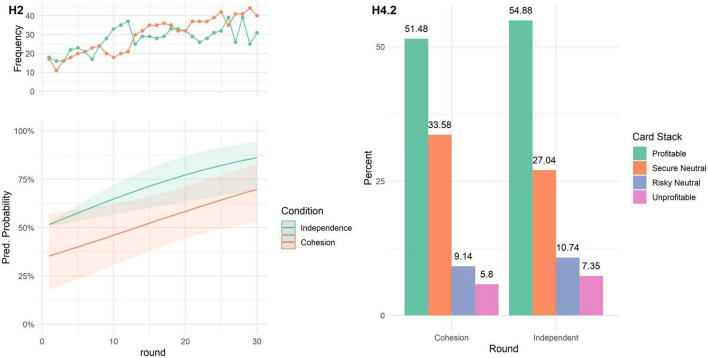
**(H2) Lower panel:** Predicted probability of choosing the profitable card stack in each round. Lines represent the marginal effects, shaded areas represent the 95% C.I. **Upper panel:** Raw frequency of profitable card stack choices over rounds, separate for cohesion and independent condition. **(H4.2)** Relative frequency (percentage) of card stack choices, separate for cohesion, and independent condition.

**H2**. Contrary to expectations, there was no difference in learning rates between the cohesion and independence condition. We fitted a logistic mixed model to predict choice of profitable card stack with condition (independence vs. cohesion) and round, with round, participant id, and group as random effects. The model’s total explanatory power is substantial (cond. *R*^2^ = 0.42; marg. *R*^2^ = 0.07). The results can be seen in Figure 3. The main effect of round remained significant, showing that participants in both conditions were more likely to choose the profitable card stack in later rounds (β = 0.06, 95% CI [0.03, 0.09], *p* < 0.001, std. β = 0.67), while no main effect of condition (β = −0.65, 95% CI [−1.58, 0.27], *p* = 0.167, std. β = −0.09) and no interaction effect was found (β = −0.01, 95% CI [−0.06, 0.04], *p* = 0.670, std. β = −0.26).

**H3**. Contrary to expectations, participants in the cohesion condition explored for a longer time (*M* = 13.56 rounds, *SD* = 5.23), compared to participants in the independence condition (*M* = 10.78 rounds, *SD* = 4.94). This was shown by a Welch two-sample *t*-test: *t*(106) = 0.41, *p* = 0.003, Cohen’s *d* = −0.55.

**H4**. Contrary to expectations, participants in both conditions chose the card stacks about equally well as measured by choice score (*H4.1*) and card choice (*H4.2*). There was no significant difference in choice score between conditions (*H4.1*; *M*_*Coh*_ = 73.70, SD = 7.43; *M*_*Ind*_ = 74.25, SD = 6.72); *t*(104.94) = 0.41, *p* = 0.342; Cohen’s *d* = 0.08. While there were differences between overall card stack choices (*H4.2*; Figure 3) as shown by a χ^2^-test (H4.2; χ^2^(3) = 18.231, *p* < 0.001), there was no difference between conditions in how often participants chose the profitable card stack (*p* = 1, determined by *post-hoc* tests corrected with the Bonferroni method). However, we found that participants in the cohesion condition chose the secure neutral card stack significantly more often, compared to the independent condition (*p* = 0.020).

**H5**. Contrary to expectations, the length of the exploration phase (as measured by the half-change round) did not affect the overall decision quality (as measured by the choice score). There was neither a linear nor a quadratic relationship between these two variables, as shown by a linear mixed model (estimated using REML and nloptwrap optimizer) to predict choice score with half-change round in a linear and quadratic term (formula: choice score ∼ half-change round + half-change round^2^), including group as random effect. The effect of both the linear term (β = −0.71, 95% CI [−1.69, 0.27], *t*(103) = −1.44, *p* = 0.153; std. β = −0.53, 95% CI [−1.26, 0.20]) and the quadratic term were statistically non-significant (β = 0.02, 95% CI [−0.02, 0.05], *t*(103) = 0.86, *p* = 0.390; std. β = 0.32, 95% CI [−0.41, 1.05]). However, when only including the linear term in the model, the model suggests that participants with longer exploration phases perform worse, measured by choice score (cond. *R*^2^ = 0.27, marg. *R*^2^ = 0.05; β = −0.30, 95% CI [−0.54, −0.05], *t*(104) = −2.41, *p* = 0.018; std. β = −0.22, 95% CI [−0.40, −0.04]). The model fit did not differ significantly between both models, as determined by a log likelihood test [χ^2^(1) = 0.765, *p* = 0.382].

**H6.1**. We expected that the relationship of group cohesion and decision quality as measured by the choice score would be mediated by exploration.^3^ However, no mediation effect was found. We performed a mediation analysis. The total effect (half-change round * condition on choice score) was significant (β = 2.79, 95% CI [0.36, 5.33], *p* = 0.030) as was the direct effect (β = 2.79, 95% CI [0.36, 5.33], *p* = 0.030). The indirect effect, however, remained non-significant (β = 0, 95% CI [−0.00, 0.00], *p* = 1). The proportion mediated was 0.

***H6.2***. As for H6.1, a mediation of the relationship of group cohesion and decision quality (as measured by choice of the profitable reward field) by exploration was expected. However, no mediation effect was found. While the total effect (half-change round * condition on choice of profitable card stack; β = 2.810, 95% CI [0.246, 5.07], *p* = 0.030) and the direct effect (β = 2.810, 95% CI [0.246, 5.07], *p* = 0.030) were significant, no mediation effect could be found (proportion mediated = 0) with the indirect effect (β = 0, 95% CI [−0.00, 0.00], *p* = 1) being non-significant.

## 4. Study 3

Emergent group processes (e.g., leader-/followership) were excluded in the reductionist behavioral experiment conducted in Study 2 to focus on the effects of group cohesion and exploration on group decision making. In contrast, the aim of Study 3 is to increase the complexity of the experimental design to identify which processes emerge if participants are allowed to interact through movement and how these processes might guide the group decision process. In this way, Study 3 extends the reductionist paradigm of Study 2 to the HoneyComb paradigm (Boos et al., 2019) that was previously used (Ritter et al., 2021). Doing so, Study 3 build on all previous results (Ritter et al., 2021; as well as Study 1 and 2). The group decision task was transferred to the HoneyComb paradigm for three reasons: First, the HoneyComb paradigm allows participants to interact and therefore exchange social information according to processes that have been shown for moving (animal) groups (e.g., Couzin, 2009; Moussaïd et al., 2009; Sridhar et al., 2021). Second, using the HoneyComb paradigm allows researchers to analyze the emerging group processes (Boos et al., 2014, 2019) by recording spatio-temporal data. In this way, we aim to disentangle emergent processes from each other. Third, the HoneyComb paradigm allows researchers to implement the same decision task that was used in Study 2 (e.g., using the same reward structure; see Table 1 for a comparison of study designs). In order to allow for the emergence of processes such as leadership, aspects of Ritter et al.’s (2021) study were adapted. In the current study, we allowed for a global visual radius. This means that participants can see movements of all other participants on the playfield, regardless of their distance to them. In this way, participants can sample as much social information as possible. Additionally, we used four reward fields instead of pre-programmed leaders. The differently profitable leaders were transformed into differently profitable reward fields (analogous to card stacks in Study 2, see Table 1).

In this study, we aimed to investigate the effect of group cohesion on group decision making and the mediating effect of exploration. As in Study 2 and explained in the theoretical background, we hypothesized a detrimental effect of higher group cohesion on group decision making (H1–4) and a mediating role of exploration on this effect (H5–6). The study and the following hypotheses were preregistered: 10.17605/OSF.IO/3N5RA.

*H1*: Subjects will be more likely to find the profitable field with increasing number of game rounds.

*H2*: There will be a stronger increase in the likelihood of finding the profitable field with increasing number of rounds in the independence condition, compared to the cohesion condition.

*H3*: Subjects in the cohesion condition will have a shorter exploration phase measured by the half-change round (Ritter et al., 2021), compared to those in the independence condition.

*H4.1*: Subjects will choose worse options overall, measured by the choice score (Ritter et al., 2021) in the cohesion condition, compared to those in the independence condition.

*H4.2*: Overall, subjects in the cohesion condition will choose the profitable field less often, but the safe neutral field more often compared to those in the independence condition.

*H5*: Participants with a longer exploration phase determined by the half-change round will make better decisions overall. We anticipate an inverted U-shape. We therefore expect a negative square correlation between exploration and decision quality.

*H6.1*: The relationship between condition (cohesion vs. independence) and the decision quality is mediated by the length of the exploration phase.

*H6.2*: The relationship between condition (cohesion vs. independence) and choice of the profitable field is mediated by the length of the exploration phase.

Using a post-experiment questionnaire, subjective experiences during the experiment, measures of group cohesion and entitativity (i.e., a feeling of “groupness”; Blanchard et al., 2020), and typical correlates of leadership behavior (decisiveness: Aramovich and Blankenship, 2020; achievement motivation: e.g., Karsudjono et al., 2013; self-confidence: e.g., García-Vidal et al., 2019; risk: Baškarada et al., 2017) were collected. Note that leadership behavior in the HoneyComb paradigm has not been associated with personality traits (Boos et al., 2014) as measured by the Big Five (Rammstedt and John, 2005) or agency and communion scales (Spence et al., 1979). Therefore, more behavior-oriented traits that have been shown to correlate with leadership were investigated in this study. We hypothesize that behavioral leadership as measured by a leadership score (L-F-score) will correlate with typical personality correlates of leadership (H7–10). Additionally, we expect that subjective experiences of leader-/followership (H11) and group cohesion and entitativity (H12) will correspond to behavioral measures of leadership and group cohesion. Lastly, we expect that groups will need to find effective ways of communication in earlier rounds that can be used in later rounds. We therefore expect that participants rate cooperation and interaction to be lower during earlier rounds, compared to later rounds (H13).

*H7*: There will be a positive correlation between self-confidence and leadership as measured by L-F-profiles.

*H8*: There will be a positive correlation between decisiveness and leadership as measured by L-F-profiles.

*H9*: There will be a positive correlation between profit/achievement maximization and leadership as measured by L-F-profiles.

*H10*: There will be a positive correlation between risk propensity and leadership as measured by L-F-profiles.

*H11*: There will be a positive correlation between perceived leadership/followership and leadership as measured by L-F-profiles.

*H12*: Entitativity (as measured by the mean of all group members’ reports) will be higher in the cohesion condition, compared to the independence condition.

*H13*: Cooperation and interaction (as measured by mean of all group members’ reports) in the rounds 1 through 10 will be lower than in rounds 11 through 20 and 21 through 30; cooperation and interaction in the rounds 11 through 21 will be lower than the rounds 21 through 30.

### 4.1. Methods

This study employs a mixed-design with a 2-level between-subject manipulation (independence vs. cohesion condition) with repeated measures (30 rounds).

#### 4.1.1. Sample

Data was collected from 96 participants that played the game in groups of six (i.e., 16 groups). However, one person reported a level of German below B1 and was therefore excluded from the analysis. The resulting sample consists of 95 participants (62 women, 32 men, 1 diverse; Age: *M* = 24.55 years, *SD* = 7.76). Half of the groups (*n* = 8) were assigned to the cohesion condition, the other half played the independence condition. As this study is a conceptual replication of Study 2, the same *a priori* power analysis applies (see Section “1.2. Power Simulation” in Supplementary material, additional sample information in Section “1.3.2. Study 3” in Supplementary material). All data collection procedures were approved by the Ethics Committee of the Georg-Elias-Müller-Institute for Psychology (proposal 305/2022).

#### 4.1.2. Procedure

Upon signing up to participate, participants received an invitation e-mail detailing all necessary steps to prepare their laptop for participation and a link to an online meeting room. During the experiment slot, subjects needed to use a PC or laptop to join an online video conference (BigBlueButton). In order to ensure complete anonymity between participants, participants were prohibited from sharing their camera, microphone, screen, or name. The experiment was started using an online meeting to (a) allow the experimenter to check that all participants are present and provided written consent prior to starting the game, and (b) share instructions live with participants to create social presence.

**FIGURE 4:**
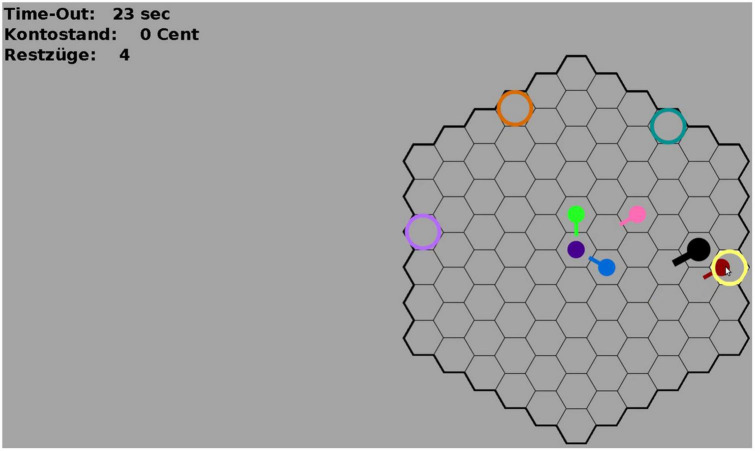
Screenshot of the HoneyComb game environment. The colored circles are the reward fields. The colored avatars are moving across the field. The avatar of the participant (black) is larger than the avatar of the other participants. Small tails on the avatars are shown for 1000 ms after each move to indicate from where the avatar had moved onto the current field. On the top-left, the remaining time in each round (“Time-Out”), the current account balance of the participant (“Kontostand”), and the remaining moves in the current round (“Restzüge”) are shown. All colors are randomly assigned at the beginning of each game and remain the same during all 30 rounds of the game (1 game = 30 rounds). Colors were chosen to be easily discriminable. Participants were naïve to the exact pay-out probabilities and had to infer the best reward field through exploration.

Participants started the game by logging into a Remote Desktop Machine on which the experiment program was running. The program is an adaptation of the HoneyComb paradigm version used by Ritter et al. (2021) and was designed to eliminate all communication channels except the visual perception of movements on the playing field. Participants were represented on the virtual playing field as colored avatars (see Figure 4) that they could move around on the playing field using mouse-clicks.

The game consisted of 30 consecutive rounds (as in Study 2) in which the rules of the game remained the same. The task for participants was to maximize their payoff by arriving at fields that yielded a monetary reward at the end of each round. There were four reward fields, represented by differently colored circles (colors were assigned randomly for each game). Participants played the game in the presence of five other participants and could observe each other’s movement behavior on the whole playing field (global visual radius). In the cohesion condition, participants received an additional reward (3 cents) for arriving on a reward field with other participants to incentivize cohesive behavior in the game. This bonus was shown to participants separately from their winnings from the reward field itself. When participants had completed the 30 game rounds, they received a link to the post-experiment questionnaire which assessed subjective experience and strategy in the game, self-confidence, decisiveness, achievement motivation, and risk propensity. Furthermore, we asked participants to rate their perception of following and/or leading others in the game, entitativity, cooperation, and interaction. After completing the questionnaire, participants were thanked and asked to leave the online conference.

#### 4.1.3. Operationalization

***Decision quality*** is operationalized analogous to Study 2.

***Group cohesion*** is operationalized in three ways. First, as the manipulated independent variable condition in which half of the groups were incentivized with a group bonus, rewarding group cohesion (cohesion condition), while the other half was not incentivized in this way (independence condition). Second, cohesion as a dependent variable is operationalized as the *average distribution* of players across the playing field. The percentage of used fields on the playing field is a measure of spatial distribution of players, a behavioral marker of group cohesion. Third, group cohesion as another dependent variable is operationalized as *clustering* in terms of the global clustering coefficient of an undirected, weighted network: In the network, players are nodes and the closeness between players (inverted numbers of fields between two players) are differently weighted edges. The global clustering coefficient of the network (transitivity) is used as a measurement for spatial clustering of players on the playing field, a behavioral marker of group cohesion in the game.

***Exploration*** is operationalized analogous to Study 2.

***Leadership*** is operationalized with the *L-F-score* of each person in each round. This score is a measure of the open behavioral aspect of leader-/followership within a group. L-F-profiles are constructed as in the following example: At the beginning of a round, all players are assigned a L-F-score of zero. Whenever a player moves during the game, the resulting difference in distance to all other members is calculated. For example, player A has moved away one field from player B and C, thereby increasing the distance to them. Player A’s L-F-score is increased by 1 point, players’ B and C score is decreased by 1 point. Next, player B moves closer to player A, but away from player C. Again, player A’s L-F-score is increased by 1 point (someone else “followed” them). Player B’s L-F-score remains the same (moved away from C, but closer to A) and player C’s score is further decreased by 1. For every move, the L-F-score for all players is updated. For each new round, a new L-F-score is calculated that is summed up to calculate the *overall L-F-score* measuring leadership over all 30 rounds.

***Self-confidence*** was measured using the Multidimensionale Selbstwertskala (internal consistency: Cronbach’s α = 0.76 −0.87; retest reliability: *r*_*tt*_ = 0.69–0.82; Schütz et al., 2016). ***Decisiveness*** was measured using the Decisiveness Scale (internal consistency: Cronbach’s α = 0.82 −0.87; Roets and Van Hiel, 2007). ***Achievement motivation*** was measured using the short version of the Leistungsmotivationsinventar (internal consistency: Cronbach’s α = 0.68 −0.86; retest reliability: *r*_*tt*_ = 0.66 −0.82; Schuler and Prochaska, 2001). ***Risk propensity*** was measured with the R-1 measure (retest reliability: *r*_*tt*_ = 0.64; Beierlein et al., 2014). Additionally, two scales assessed ***entitativity*** (Gaertner and Schopler, 1998; Blanchard et al., 2020). All scales were validated and measurement criteria can be found in the cited original articles or manuals.

#### 4.1.4. Data preprocessing and analysis

Data was preprocessed and analyzed as described in the preregistration^4^ using R, running in RStudio (R Core Team, 2020; RStudio Team, 2020), using the packages cited in the Section “1.4. R packages” in Supplementary material. The analysis script and preprocessed data can be found on the OSF project (see text footnote 1).

### 4.2. Results

#### 4.2.1. Confirmatory analyses

Confirmatory analyses were run as described in the preregistration and are presented in order of the hypotheses.

**Manipulation check**. The manipulation check was successful. A one-sided Wilcox Rank–Sum Test with continuity correction, used since variables violated assumptions of normality, showed that groups in the cohesion condition used significantly fewer fields (*Mdn* = 12.14% of all fields) than groups in the independence condition (*Mdn* = 20.86%); *W* = 2268, *p* < 0.001. The same holds for the field distribution calculated on the move level (*Mdn*_*Ind*_ = 4.90%; *Mdn*_*Coh*_ = 4.07%; *W* = 2268, *p* < 0.001) and clustering (*Mdn*_*Ind*_ = 0.80, *Mdn_*Coh*_* = 0.85, *W* = 0, *p* < 0.001).

***H1***. As predicted, participants were more likely to choose the profitable reward field in later rounds. We fitted a logistic mixed model (estimated using ML and BOBYQA optimizer). In the model the choice of the profitable field could be predicted with round (formula: choice of field ∼ −1 + round). The model included round, participant id and group as random effects. The model’s total explanatory power is substantial (conditional *R*^2^ = 0.66) and the part related to the fixed effects alone (marginal *R*^2^) is 0.06. The effect of round is statistically significant and positive (β = 0.09, 95% CI [0.04, 0.14], *p* < 0.001; std. β = 0.47, 95% CI [0.28, 0.67]). Standardized parameters were obtained by fitting the model on a standardized version of the dataset. 95% Confidence Intervals (CIs) and *p*-values were computed using a Wald z-distribution approximation. All following models were fitted using the same method if not stated otherwise.

**FIGURE 5:**
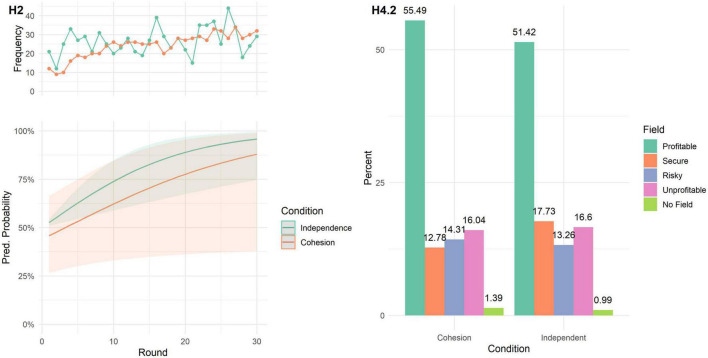
**(H2) Lower panel:** Predicted probability of choosing the profitable field in each round. Lines represent the marginal effects, shaded areas represent the 95% C.I. **Upper panel:** Raw frequency of profitable field choices over rounds, separate for cohesion and independent condition. **(H4.2)** Relative frequency (percentage) of reward field choices, separate for cohesion and independent condition.

***H2***. Contrary to expectations, participants in both conditions were more likely to choose the profitable field in later rounds and there was no difference between conditions in this effect as can be seen in Figure 5. This was shown by a logistic mixed model, predicting choice of the profitable field with condition and round (fixed effects) and including round, participant id, and group as random effects. The model’s total explanatory power is substantial (cond. *R*^2^ = 0.68) and the marginal *R*^2^ is 0.07. The effect of condition is statistically non-significant (β = −0.24, 95% CI [−1.08, 0.60], *p* = 0.570, std. β = 0.40), and the effect of round is statistically significant and positive (β = 0.11, 95% CI [0.04, 0.17], *p* = 0.002, std. β = 0.46). The interaction effect is statistically non-significant (β = −0.03, 95% CI [−0.13, 0.07], *p* = 0.535, std. β = 0.009).

***H3***. Contrary to expectations, no differences in the length of exploration phases were found between conditions, as shown by a one-sided Wilcox Rank–Sum Test with continuity correction. In both the cohesion and independence condition, the half-change round median was at 14 rounds (*M*_*Coh*_ = 11.21 rounds, *M*_*Ind*_ = 12.48 rounds); *W* = 1254, *p* = 0.776.

***H4.1***. Contrary to expectations, participants in both conditions scored about equally well on the choice scores. As shown by a Wilcoxon-signed rank test, the median choice scores for the cohesion and independence condition (*M*_*Ind*_ = 70.35 points, *M*_*Coh*_ = 71.52 points) did not differ significantly; *W* = 1095.5, *p* = 0.662.

***H4.2***. Contrary to predictions, there was no difference between the number of times participants in the cohesion condition chose the profitable reward field, compared to participants in the independent condition (*post hoc p* = 1, Bonferroni corrected; Figure 5). Further, participants in the cohesion condition chose the secure neutral field less often, compared to the independence condition (*post hoc p* = 0.023, Bonferroni corrected); χ^2^(4) = 16.496, *p* = 0.002.

***H5***. Contrary to expectations, a medium exploration length was associated with lower choice scores, while shorter and longer exploration lengths were associated with higher choice scores. We fitted a linear mixed model (estimated using REML and nloptwrap optimizer) to predict the choice score with the length of the exploration phase (half-change round) in a linear and quadratic term (formula: choice score ∼ half-change round + half-change round^2^). The model included group as a random effect. The model’s total explanatory power is substantial (cond. *R*^2^ = 0.65) and the part related to the fixed effects alone (marg. *R*^2^) is of 0.42. The effect of the linear term is statistically significant and negative (β = −2.88, 95% CI [−4.27, −1.49], *t*(91) = −4.11, *p* < 0.001; std. β = −1.29, 95% CI [−1.92, −0.67]). The effect of the quadratic term is statistically significant and positive (β = 0.08, 95% CI [0.02, 0.14], *t*(91) = 2.57, *p* = 0.012; std. β = 0.74, 95% CI [0.17, 1.31]). This model was compared to a model using only the linear term: This model’s total explanatory power was substantial (cond. *R*^2^ = 0.60; marg. *R*^2^ = 0.32). The effect of the liner term is statistically significant and negative (β = −1.13, *95% CI* [−1.50, −0.76], *t*(92) = −6.13, *p* < 0.001, std. β = −0.51). The quadratic model outperformed the linear model significantly in a Log-Likelihood comparison [χ^2^(1) = 6.62; *p* = 0.010] and on all model performance indices (RMSE, Sigma, AIC, BIC, cond. *R*^2^, marg. *R*^2^), except for the ICC (Supplementary Figure 1).

***H6.1***. Contrary to expectations, exploration did not mediate the relationship of cohesion and decision quality as measured by the choice score. A mediation analysis was performed, even though the general effect (condition – choice score) was not found. The total effect of condition on choice score (β = −1.37, 95% CI [−5.77, 2.92], *p* = 0.520) was non-significant as were the direct effect (β = −1.37, 95% CI [−5.77, 2.92], *p* = 0.520) and the indirect effect (β = 0, 95% CI [−0.00, 0.00], *p* = 1). The proportion mediated was 0.

***H6.2***. Contrary to expectations, exploration length did not mediate the relationship of group cohesion on group decision making as measured by choice of profitable reward field. No mediation effect could be found (proportion mediated = 0) with the total (β = −1.33, 95% CI [−5.75, 2.74], *p* = 0.520), direct (β = −1.33, 95% CI [−5.75, 2.74], *p* = 0.520), and indirect effect (β = 0, 95% CI [−0.00, 0.00], *p* = 1) being non-significant.

***H7-H11***. Contrary to expectations, the L-F-Scores did not correlate with self-confidence, achievement motivation, decisiveness, or risk propensity. However, the data showed that participants who reported following others, were more likely to have lower L-F-scores (indicating followership). Further, participants with higher (vs. lower) L-F-scores were also more likely to report taking a leading (vs. following) role, and vice versa. Results of the correlation analyses can be seen in Table 2.

**TABLE 2:** Correlation results of leadership scores.

	1	2	3	4	5	5.1	5.2	5.3	5.4	6	7
**2**	-0.199										
**3**	0.269*	0.219									
**4**	0.324**	-0.313**	0.501***								
**5**	0.018	-0.024	0.073	0.091							
**5.1**	0.037	0.033	0.048	0.066	0.882***						
**5.2**	0.053	-0.183	0.019	0.131	0.749***	0.610***					
**5.3**	-0.013	0.038	0.093	0.074	0.846***	0.623***	0.486***				
**5.4**	-0.007	-0.024	0.066	0.034	0.793***	0.652***	0.464***	0.543***			
**6**	0.094	-0.117	0.162	0.102	0.338***	0.371***	0.312**	0.111	0.380***		
**7**	0.054	0.217*	0.032	-0.145	-0.368***	-0.247***	-0.250*	-0.401***	-0.278***	0.05	
**8**	0.131	-0.15	0.218	0.142	0.174	0.127	0.193	0.189	0.05	0.206***	-0.035

Pearson correlation coefficients (*r*) of associations between observed leadership behavior (1), self-reported leadership behavior (2–4), and typical leadership correlates (5–7). 1–L-F-scores, 2–self-reported followership, 3–self-reported leadership, 4–self-reported role (1 = follower, 7 = leader), 5–self-confidence, 5.1–emotional self-esteem, 5.2–social self-esteem in contact with others, 5.3–social self-esteem when critiqued, 5.4–performance-related self-esteem, 6–achievement motivation, 7–decisiveness, 8–risk propensity.

*p < 0.05, **p < 0.01, ***p < 0.001.

***H12***. As expected, participants in the cohesion condition reported significantly higher levels of entitativity, compared to participants in the independence condition (*M*_*Coh*_ = 4.38, *M*_*Ind*_ = 2.73). A Welch Two-Sample *t*-test showed a positive and large effect: *t*(91.46) = 7.17, *p* < 0.001; Cohen’s *d* = 1.46, 95% CI [1.01, 1.91].

**FIGURE 6:**
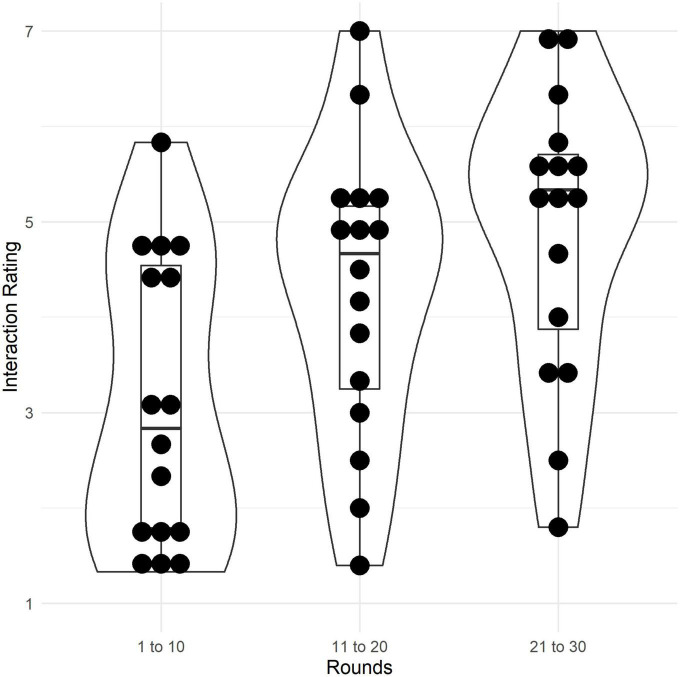
Ratings of interactivity (retrospective self-report) during the first (round 1–10), second (11–20), and last third (21–30) of the game (7-point Likert scale). Violin plots show density distributions of ratings. Boxes represent the interquartile range, the thick line in the box represents the median, whiskers represent the range of data, dots represent raw data.

***H13***. As expected, participants reported that interaction increased over rounds (Figure 6). Three paired *t*-tests (Bonferroni corrected) showed that participants rated interaction higher in rounds 11–20 (*M* = 4.30), compared to rounds 1–10 [*M* = 3.14; *t*(95) = −6.81, *p* < 0.001]; Cohen’s *d* = −0.70, 95% CI [−0.92, −0.47]. Interaction in rounds 21–30 (*M* = 4.92) was rated even higher, compared to both rounds 1–10 [*t*(95) = −9.27, *p* < 0.001; Cohen’s *d* = −0.95, 95% CI [−1.19, −0.70]] and rounds 11–20 [*t*(95) = −5.48, *p* < 0.001; Cohen’s *d* = −0.56, *95% CI* [−0.77, −0.34]].

#### 4.2.2. Exploratory results

In our previous study (Ritter et al., 2021), a major methodological drawback was the low correlation between the choice score and participants’ earnings. This problem seems to have been eliminated in the current study: We fitted a linear mixed model to predict earnings with condition and choice score, including group as a random effect (cond. *R*^2^ = 0.97, marg. *R*^2^ = 0.87). While the effect of condition was non-significant [β = 117.19, 95% CI [−151.44, 385.81], *t*(90) = 0.87, *p* = 0.388, std. β = 1.31], choice score was positively associated with earnings [β = 12.58, 95% CI [11.19, 13.98], *t*(90) = 17.92, *p* < 0.001, std. β = 0.56]. No significant interaction could be found [β = 3.18, 95% CI [−0.39, 6.75], *t*(90) = 1.77, *p* = 0.080, std. β = 0.14].

We explored whether participants could indicate explicitly which reward field had been the most profitable one. We found that 70.5% of all participants were able to identify the profitable field explicitly with a self-reported certainty of 57.1% on average (*SD* = 30.76). Those participants who did not correctly identify the profitable field reported a significantly lower certainty (*M* = 27.5%, *SD* = 18.86); *t*(79.95) = −5.71, *p* < 0.001; Cohen’s *d* = −1.16, 95% CI [−1.59, −0.72].

We further explored the correlations between the measures of cohesion on the HoneyComb field (condition, field distribution, transitivity) and subjective cohesion measures. Results can be found in Table 3. Significant correlations were found between virtually all cohesion measures, except for ratings of similarity within a group and some interaction ratings. Notably, we found a significant medium association between cohesion (as measured by field distribution) and decision quality; *r* = −0.26, 95% CI [−0.44, −0.07], *t*(93) = −2.64, *p* = 0.010.

**TABLE 3:** Correlation results of group cohesion scores.

	1	2	3	4	5	6	7	8	9	10	11
**2**	0.866***										
**3**	-0.799***	-0.862***									
**4**	-0.842***	-0.915***	0.967***								
**5**	0.650***	0.656***	-0.608***	-0.655***							
**6**	0.153	0.189	-0.239***	-0.233***	-0.026						
**7**	-0.632***	-0.646***	0.660***	0.666***	-0.601***	-0.436***					
**8**	0.595***	0.590***	-0.589***	-0.616***	0.598***	0.113	-0.637***				
**9**	0.559***	0.583***	-0.591***	-0.597***	0.614***	0.239*	-0.653***	0.654***			
**10**	0.039	0.09	-0.153	-0.149	0.249***	0.082	-0.233***	0.280***	0.471***		
**11**	0.557***	0.590***	-0.548***	-0.585***	0.552***	0.281***	-0.673***	0.577***	0.719***	0.453***	
**12**	0.342***	0.362***	-0.359***	-0.372***	0.217***	0.19	-0.296***	0.443***	0.413***	0.261***	0.381***

Person correlation coefficients (*r*) of associations between manipulated (1), observed (2–4), and self-reported cohesion and entitativity measures. 1–condition, 2–transitivity, 3–field distribution, 4–field distribution (move level), 5–interaction rating (start of the game), 6–interaction rating (during the game), 7–interaction rating (none), 8–entitativity, 9–entitivity, 10–similarity, 11–interactivity, 12–goals. All correlations are Pearson correlation coefficients (with all correlations with condition being point-biserial correlations).

*p < 0.05; **p < 0.01; ***p < 0.001.

**FIGURE 7:**
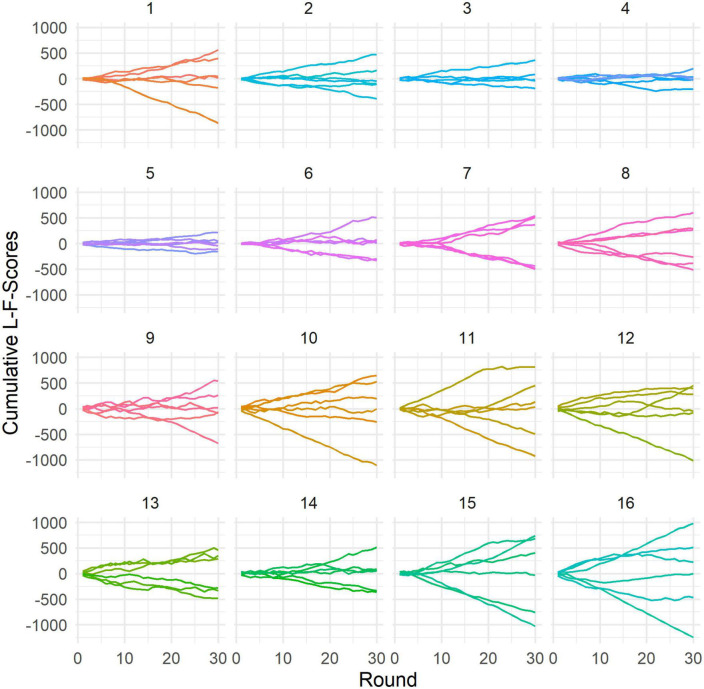
Cumulative L-F-score sums over rounds. Each panel shows data from one group. Each line represents the cumulative L-F-score sum of one individual participant. Higher scores represent leadership behavioral patterns, while lower scores represent followership behavioral patterns. More divergent cumulative L-F-score sums indicate spontaneous emergence of leader-/follower roles within a group.

Lastly, we explored whether we could find evidence that leader-/followership emerged over interactions. Leader and follower roles seem to have emerged in most groups (Figure 7), except for groups 3, 4, and 5. Notably, a significant difference in decision quality can be found between those three groups (choice score: *M* = 64.65, *SD* = 9.71) and groups in which leader-/followership emerged (*M* = 72.62, *SD* = 11.37); *t*(93) = −2.68, *p* = 0.009; Cohen’s *d* = −0.72, 95% CI [−1.25, −0.18]. However, there was no significant correlation between decision quality and leadership (as measured by L-F-scores); *r* = 0.007, 95% CI [−0.19, 0.21], *t*(93) = 0.07, *p* = 0.946.

## 5. Discussion

The aim of this paper was to provide detailed insights into the relationships between group cohesion, length of exploration phases, and group decision making under uncertainty and the processes that might drive these relationships. Specifically, we conducted three studies with increasing complexity: Study 1, a simulation study, identified suitable cohesion manipulations and investigated how differing lengths of exploration would affect group decision making. Study 2 examined in a behavioral experiment how group cohesion can affect exploration lengths and the quality of group decision making when only limited social information is available. Study 3 used a group movement paradigm to identify and disentangle group processes that emerge in group decision making under uncertainty and to study how these processes might drive the relationships between group cohesion, exploration, and the quality of group decision making.

Specifically, we used the ε-greedy algorithm in Study 1 to identify a new cohesion reward (additive bonus) that was implemented in the empirical studies (Study 2 and 3). In the second study, the effect of group cohesion on exploration and group decision making was tested in a card-choice task. Here, group cohesion was manipulated experimentally by rewarding group members choosing the same option with an additive group bonus. In the third study, we implemented the task within the HoneyComb paradigm (Boos et al., 2019) that allows participants to interact or communicate with each other *via* movement, while the emerging group processes can be observed in detail *via* analysis of the spatio-temporal data. In this way, we were able to identify which group processes emerged during group decision making under uncertainty (leadership, group cohesion) and how these processes might have affected the decision outcome.

The results of Study 1 corroborated claims that a multiplicative reward structure might not be suitable in group experiments. Results of the simulation indicate that successful inference of the best option is much harder with a multiplicative cohesion reward as the multiplication of rewards creates non-stationary distributions. This is in line with previous findings (Ritter et al., 2021). With a multiplicative reward, each time agents in a simulation (Study 1) or human participants (Study 2 and 3) choose a certain option, the reward attributed to the option is obfuscated by the number of agents choosing the same option. This has been coined unexpected uncertainty (Cohen et al., 2007) or even volatility (Bland and Schaefer, 2012). In comparison, simulations using an additive cohesion reward fared better and simulations using no cohesion reward were the fastest to find the optimal solution (Study 1). It should be noted that including an additive cohesion reward will still violate the assumption of stationary distributions. However, with a small additive reward, the problem of non-stationarity is less pronounced as the actual option reward remains the dominant feedback. The additive bonus was implemented in Studies 2 and 3. Additionally, the separation of the feedback of the reward and cohesion bonus was introduced. In this way, we could empirically support the proposal that empirical studies can use additive cohesion rewards without hampering a group’s ability for collective induction. That even an additive group bonus can successfully manipulate group cohesion was shown in Study 3 where groups receiving the additive bonus showed greater observed, spatial cohesion as well as self-reported cohesion and entitativity.

Additionally, Study 1 showed that how much agents explore during the decision task (as represented by the ε parameter) will strongly impact performance. Lower exploration rates (1 and 10% of trials used for exploration) produced suboptimal results in which agents were slower to identify the optimal solution. In contrast, higher exploration rates (20 and 50%) in the simulations led to agents finding the optimal solution more quickly. As an outlier to this general pattern, the simulation using ε = 0.05 performed about as good as the simulation with ε = 0.5. Future simulation studies might investigate whether this is a systematic effect or, more likely, a random finding. After all, the amount of trials that agents use for exploration are determined randomly. Additionally, future simulations might investigate whether ceiling effects of exploration exist. The results of Study 1 suggest that higher exploration rates will lead to better optimization results. However, it seems implausible that this would generalize to other simulations or real-world decisions, as there is usually a trade-off between exploration and exploitation (e.g., Cohen et al., 2007; Tickle et al., 2021). In fact, this inverted U-shape (high performance using a medium ε) is frequently found in applications of the ε-greedy algorithm (Sutton and Barto, 2018). It could be that adapting the simulation to include only 30 iterations (corresponding to the 30 rounds in the human experiment) allowed more room for erroneous convergence.

Findings from Study 1 were used to eliminate previous methodological limitations to design behavioral experiments on human group behavior focusing on (a) the use of social information in group decision making in a reductionist experiment paradigm (Study 2), and (b) emergent group processes guiding group decision making under uncertainty in a movement-based behavioral experiment (Study 3). In both experimental studies, groups were able to identify the best option (Study 2: card stack, Study 3: reward field; *H1*) as was also found in previous research (Ritter et al., 2021). However, no difference between conditions could be found in terms of decision quality (*H2*, *H4*): In both studies, groups that were rewarded for cohesion chose the best option about equally as often as groups who were not rewarded for cohesion. Learning rates (i.e., increasing probability of choosing the best option with increasing rounds) did not differ between conditions either. By extension, groups in both conditions performed equally well in terms of the overall decision quality (measured by the choice score), in both studies. It should be noted that the absence of a significant difference should not be interpreted as evidence for the absence of an effect. Instead this lack of effect should be investigated in the future or analyzed using Bayesian statistics to determine whether the evidence supports the null hypothesis.

Contrary to expectations, group cohesion did not shorten participants’ exploration phases (*H3*). While participants in both conditions explored for an approximately equal length of time in Study 3, participants in the cohesion condition actually spent longer on exploration in Study 2. This difference in exploration was not found previously (Ritter et al., 2021) and could originate in the design of the study: In order to get the cohesion reward in Study 2, participants had to choose the same cards as the other group members. As there was no possibility to communicate, this was no simple task: Participants could not simply follow the tendencies of other group members (as in Study 3) but had to take a chance by switching their card stack, hoping that the other group member will choose the same card in the next round as well. Due to the limitation of communication possibilities, participants might have “missed” each other, creating a need to switch choices again in the next round. With the chosen operationalization (half-change round), choice switches due to exploration motivations cannot be distinguished from choice switches serving cohesion purposes.

We predicted that a medium level of exploration will lead to the highest decision quality, while lower and higher exploration levels will decrease decision performance. Results of Study 3 show the opposite relationship with medium exploration rates being associated with the lowest choice scores, while low and high exploration rates were associated with higher choice scores (*H5*). In Study 2, no relationship between exploration and decision quality was found. Additionally, the hypothesized mediation of group cohesion on decision quality through exploration (*H6*) could not be shown in either Study 2 or 3. As the relationship between exploration and decision quality was so surprising and contradictory to previous findings (Sutton and Barto, 2018; Yahosseini et al., 2018), we suspect that the task might have been too simple (i.e., a ceiling effect of decision performance). In adapting the paradigm by Ritter et al. (2021), care was taken to eliminate distracting information in order to gain as much insight as possible into the basic mechanism of group decision making under uncertainty. The main methodological limitation of the previous study (i.e., occlusion of information due to the cohesion reward) was successfully eliminated as could be shown by the high association of earned rewards and decision quality in both conditions. As an additional adaptation, the expected value of the profitable option was made more distinct from the expected value of the neutral options (Table 1). The clear distinction between options will have reduced uncertainty in Study 2 and 3. Additionally, the local visual radius in the previous study (Ritter et al., 2021) allowed participants to observe the behavior of other group members only when they were close to them. With the global visual radius in Study 3 and the information about the choices of all group members in Study 2, participants had comparatively more information about the behavior of others. This means that groups in these studies could have made use of a global information transmission between members, even though previous findings mainly show local information transmission in collective information pooling (Helbing et al., 2000; Couzin et al., 2002; Conradt and Roper, 2009; Moussaïd et al., 2009). With this large amount of information to go on in a less uncertain environment, participants needed only little exploration before being able to identify the most profitable option. Once participants had identified the best option, they could transition from exploration of options to exploitation of the best. This seems even more likely as the predicted probability of choosing the profitable field in early rounds (see Figures 3, 5) is already higher (>35%) than predicted by chance alone (25%).

In summary, Study 2 and 3 could replicate the previously found effect (Ritter et al., 2021) that groups can identify the best option under uncertainty, while differences between cohesive groups and groups with independent members could not be found. It is for future research to decide whether this lack of differences is due to methodological limitations of the presented empirical studies or whether group cohesion simply does not significantly impact uncertainty reduction in human groups.

The main goal of Study 3 was to identify and disentangle the group processes that might emerge to reduce uncertainty in group decision making. To this end, we explored whether behavioral measurements of two specific processes, emerging leader-/followership patterns and entitativity, would correspond to self-reported participant experience and typical correlates of these group processes. Results showed that leader-/followership as operationalized by the L-F-score was positively associated with self-reported experience of leading or following. Yet, typical trait correlates of leadership behavior (self-confidence, achievement maximization, decisiveness, risk propensity) were not associated with leadership in this study. This corresponds to previous findings: Boos et al. (2014) found leadership behavior in the HoneyComb paradigm was not associated with the Big-Five personality factors and recent work showed that links between personality and leadership are highly conditioned on the group or team context (Mitchell et al., 2022). Notably, we could show that leadership and followership emerged in most groups in Study 3, regardless of experimental condition. A first analysis suggests that leaderless groups (i.e., groups in which leader-/followership did not emerge) performed worse in terms of decision quality. This is in line with findings that the emergence of leadership can have an advantageous effect in group movement and group decision making (Boos et al., 2014; Ioannou et al., 2015; Strandburg-Peshkin et al., 2015; Sridhar et al., 2021; von Ameln, 2021). However, as most groups in our study spontaneously exhibited leader-/followership, this result can be only preliminary in nature.

In terms of group cohesion, Study 3 suggests that behavioral cohesion as measured by spatial distribution on the HoneyComb playing field (field distribution) is closely associated with self-reported group cohesion measures, such as entitativity, interactivity, and shared goals. Manipulation of group cohesion through implementing a cohesion reward also proved to be successful. Importantly, participants in groups with higher behavioral cohesion (field distribution) were also more likely to make better decisions, regardless of condition. This finding is contrary to previous findings (Ritter et al., 2021) and shows the importance of future research investigating the effect of cohesion on group decision making. Overall, these are encouraging findings as they indicate that behavioral measures of group cohesion and leader-/followership can be used to identify basic processes of group behavior and, perhaps, even predict group performance in decision making under uncertainty.

In summary, this paper contributes to the theoretical understanding of group decision processes under uncertainty by identifying key elements of the decision task as well as emergent group processes (i.e., group cohesion, leadership) that contribute to the decision outcome. While the theoretical relationship of cohesion and group decision outcomes could not be solved conclusively, this paper was able to identify non-stationarity of a decision problem (see Study 1) as an important determinant of decision outcomes. This has methodological implications as future group decision studies should be designed with this finding in mind. Additionally, this paper could show that emergent group processes such as leadership spontaneously emerge in a reductionist experimental group movement paradigm and that these processes might play a role in group decision making under uncertainty. Moreover, findings in this paper indicate that behavioral patterns of group cohesion and leadership correspond to subjective reports of participants, paving the way for the development of behavioral markers of these processes.

### 5.1. Limitations and future directions

The main limitation of this paper is the low difficulty of the group decision task used in Study 2 and 3. In choosing to adapt the task from a previous work (Ritter et al., 2021), the uncertainty inherent in the task was reduced so much as to create a ceiling effect of performance in the task. Adaptations directed at reducing uncertainty in the current studies might have overshot their goal and future research should determine whether the expected relationships present themselves in a study with medium uncertainty. Future adaptations should adjust the expected values associated with the reward choices and, possibly, restrict the visual radius of the HoneyComb paradigm to a local visual radius again in which participants can only observe the behavior of group members in their close vicinity. In this way local information transmission between group members (e.g., Conradt and Roper, 2005) might be investigated more closely. In addition, future simulation studies might be used to determine a well-balanced design of different choice options so that experimental studies can be designed accordingly.

Additionally, the studies in this paper manipulated only two levels of group cohesion. While group cohesion was not incentivized in the independence condition, spontaneous group cohesion emerged (see Study 3), albeit it was lower than in the cohesion condition. Future studies might investigate three levels of group cohesion (e.g., none, medium, and high) in order to investigate the claim that “right balance of interdependence and independence” (Conradt, 2012, p. 1) might be crucial for group decision making.

While this paper was able to provide first insights into processes that groups might use while making decisions under uncertainty (i.e., cohesive behavior, leader-/followership, collective information pooling), the three presented studies were not able to clearly differentiate between these processes. Even more so, the experimental paradigm might not be able to clearly distinguish the contributions of environmental factors and social information (Mehlhorn et al., 2015). Future studies might address this issue, for example, by using different operationalizations (e.g., centrality of group networks to classify leadership, Lusseau and Conradt, 2009; first-mover classification, Boos et al., 2014) or analysis methods (testing differences between cohesion and their effect on decision making, Casey-Campbell and Martens, 2009; applying time-dependent cross-correlations, Lombardi et al., 2020).

The samples recruited for Study 2 and 3 consisted mainly of German psychology students and are, therefore, not representative of the general population. We argue that the processes investigated in this paper are very basic, as also argued by previous research (e.g., Belz et al., 2013), and can also be observed in some primate groups (e.g., Conradt et al., 2009). To the best of the authors’ knowledge, there is little to no evidence suggesting that the observed processes might differ significantly across cultures. Furthermore, previous research (Boos et al., 2014) as well as findings of Study 3 suggest that interindividual differences are not associated with behavior in the HoneyComb paradigm. However, future research should confirm this assumption by comparatively testing our results across representative samples from different backgrounds or specifically focusing on the influence of interindividual characteristics on group decision making. For example, different propensities to explore or follow others’ decisions might be incorporated in simulation studies similar to Study 1 by furnishing agents with different behavioral patterns or parameters.

In order to design future studies, simulations of group decision making, as in Study 1, should be used to determine parameters or principles that might guide group decision making or group movement (e.g., Hornischer et al., 2022). First, the basic ε-greedy algorithm that was used to model behavior in the experiment might be adapted to include (a) the “soft max decision rule” or (b) a combination of the soft max decision rule and an “uncertainty bonus” (Cohen et al., 2007). Using the soft max decision rule, options are chosen with a probability weighted by their estimated values. This means, as in the ε-greedy algorithm, agents preferentially choose the option with the highest value. However, this rule is “softened” by the relative value of other options and noise added to the decision rule (i.e., even when a decision rule would dictate exploitation of an option, agents might randomly choose another option to explore). Lastly, the uncertainty bonus expands the soft max decision rule by promoting exploration of previously unchosen options. This is implemented by increasing the probability of choosing options that have not been explored by the uncertainty bonus, thereby driving exploration. Another possibility is to include a function with which the exploration parameter ε decreases over time, in order to model findings on human exploration behavior (e.g., Bechara et al., 1994) more closely. Using these adaptations of the ε-greedy algorithms has been shown to be more suited to predict behavior under unexpected uncertainty or volatility (i.e., when rewards might vary above and beyond the known uncertainty of an option; Cohen et al., 2007; Bland and Schaefer, 2012). In this way, these algorithms might be invaluable in investigating how exploration/exploitation decisions are made by groups. Second, future simulation studies should aim to model mechanisms that balance personal preferences and goal-orientation with social motivations (e.g., Sridhar et al., 2021) in order to reflect different motivations guiding decisions of individual group members. Third, the refinement of the ε-greedy algorithm can be informed by studying or including other decision models, such as Bayesian SPRT, drift-diffusion, or adaptive gain models to determine stopping rules for exploration (Tickle et al., 2021). Another possibility might be to use Bayesian network decision making models that incorporate agents holding private information and leveraging this for collective decisions (Hązła et al., 2021).

In summary, we argue that future research investigating group decision making under uncertainty should start with simulations in order to pin-point key variables and parameters within this opaque process. In this way, empirical studies can be designed to specifically assess, manipulate, or control the effect of these variables in empirical investigations of human behavior. This approach has two advantages: First, simulations can include a wide range of variables, test boundary conditions, and reveal relationships that can generate new hypotheses. Second, using simulations first and designing targeted experiments based on them will direct researchers’ resources at promising relationships, instead of running a series of costly experiments.

## 6. Conclusion

In this paper, we replicate findings showing that groups are able to successfully cope with an uncertain environment. Contrary to previous findings (Ritter et al., 2021), groups performed well in the decision task even when they were rewarded for cohesive behavior, and longer exploration times were not detrimental to group decision making. Future research will have to assess whether these findings hold when more uncertainty is introduced into the group decision task. We were able to provide first results that both behavioral (vs. manipulated) cohesion and leader-/followership might contribute to uncertainty reduction in groups and might be identified using behavioral markers. Future research might use methodological insights from this paper on the interchange of computer simulation and experimental design to further investigate the interrelations of group cohesion, exploration, and leadership in group decision making under uncertainty. For now, we conclude that when the environment is laid out in no uncertain terms, group cohesion will not deter groups from exploring their options and making profitable decisions.

## Data availability statement

The datasets presented in this study can be found in online repositories. The names of the repository/repositories and accession number(s) can be found below: https://s.gwdg.de/y0cOIu [project site on the Open Science Framework (OSF)].

## Ethics statement

The studies involving human participants were reviewed and approved by the Ethics Committee of the Georg-Elias-Müller-Institute for Psychology University of Göttingen, Göttingen, Germany. The patients/participants provided their written informed consent to participate in this study.

## Author contributions

MR, JP, and MB contributed to the conception and design of all three studies, and revised the manuscript. MR and LM were responsible for writing of the preregistration of Study 3. JP was responsible for data collection in Study 1, programmed the simulation study, and the HoneyComb paradigm. EB programmed the online experiment in Study 2. EB and LM were responsible for data collection in Study 2 and 3, respectively. MR conducted all data analyses and wrote the first draft of the manuscript. MR and MB were responsible for scientific supervision. All authors read and approved the submitted version.

## Footnotes

1
https://s.gwdg.de/y0cOIu

2 We recalculated all mediation analyses (H6.1 and H6.2 in Study 2 and 3) using a quadratic term for exploration. However, the results remained the same so that we report the less complex models.
