# Supplementary material for: In no uncertain terms: Group cohesion did not affect exploration and group decision making under low uncertainty
Source: Front Psychol. 2023 Jan 25;14:1038262. doi: 10.3389/fpsyg.2023.1038262 (PMC9905233; doi:10.3389/fpsyg.2023.1038262)
Supplement: Supplementary file 1 [file Data_Sheet_1.pdf]

Supplementary Material

1 Supplementary Material

1.1 Additional information on regression analyses

1.1.1 Study 1

1.1.1.1 Model predicting the number of agents on profitable option with round and group bonus type

We fitted a Poisson mixed model (estimated using ML and BOBYQA optimizer) to predicting the number of agents choosing the profitable option with group bonus type and round (formula: Profitable ~ bonus * round). The model included simulation run as a random effect (formula: ~1 | run). The model's total explanatory power is substantial (conditional R^2 = 0.57) and the part related to the fixed effects alone (marginal R^2) is of 0.05. The model's intercept, corresponding to bonus type = Additive and round = 0, is at 0.51 (*95% CI* [0.47, 0.56], *p* < .001). Within this model:

- The effect of bonus [Multiplicative] is statistically non-significant and negative (β = -0.05, *95% CI* [-0.11, 0.01], *p* = .136; std. β = -0.10, *95% CI* [-0.16, -0.04]).

- The effect of bonus [None] is statistically significant and positive (β = 0.11, *95% CI* [0.05, 0.17], *p* < .001; std. β = 0.13, *95% CI* [0.07, 0.19]).

- The effect of round is statistically significant and positive (β = 0.02, *95% CI* [0.02, 0.02], *p* < .001; std. β = 0.18, *95% CI* [0.17, 0.18]).

- The interaction effect of round on bonus [Multiplicative] is statistically significant and negative (β = -0.003, *95% CI* [-0.004, -0.002], *p* < .001; std. β = -0.03, *95% CI* [-0.04, -0.02])

- The interaction effect of round on bonus [None] is statistically significant and positive (β = 0.001, *95% CI* [0.00, 0.002], *p* = .019; std. β = 0.01, *95% CI* [0.001, 0.02]).

Standardized parameters were obtained by fitting the model on a standardized version of the dataset. 95% Confidence Intervals (CIs) and p-values were computed using a Wald z-distribution approximation.

1.1.1.2 Model predicting the number of agents on secure neutral option with round and group bonus type

We fitted a Poisson mixed model (estimated using ML and BOBYQA optimizer) to predict the number of agents choosing the secure neutral option with bonus type and round (formula: SecureNeutral ~ bonus * round). The model included run as a random effect (formula: ~1 | run). The model's total explanatory power is substantial (conditional R^2 = 0.55) and the part related to the fixed effects alone (marginal R^2) is of 0.005. The model's intercept, corresponding to bonus = Additive and round = 0, is at 0.19 (*95% CI* [0.13, 0.24], *p* < .001). Within this model:

- The effect of bonus [Multiplicative] is statistically significant and positive (β= 0.08, *95% CI* [0.003, 0.16], *p* = .041; std. β = 0.15, *95% CI* [0.08, 0.23])

- The effect of bonus [None] is statistically significant and positive (β= 0.21, *95% CI* [0.13, 0.29], *p* < .001; std. β = 0.15, *95% CI* [0.08, 0.23])

- The effect of round is statistically significant and negative (β= -0.003, *95% CI* [-0.004, -0.002], *p* < .001; std. β = -0.03, *95% CI* [-0.04, -0.02])

- The interaction effect of round on bonus [Multiplicative] is statistically significant and positive (β = 0.004, *95% CI* [0.003, 0.005], *p* < .001; std. β = 0.04, *95% CI* [0.03, 0.05])

- The interaction effect of round on bonus [None] is statistically significant and negative (β = -0.003, *95% CI* [-0.005, -0.002], *p* < .001; std. β = -0.03, *95% CI* [-0.05, -0.02])

Standardized parameters were obtained by fitting the model on a standardized version of the dataset. 95% Confidence Intervals (CIs) and p-values were computed using a Wald z-distribution approximation.

1.1.1.3 Model predicting the number of agents on profitable option with round and epsilon parameter

We fitted a Poisson mixed model (estimated using ML and BOBYQA optimizer) to predict the number of agents choosing the profitable option with Epsilon and round (formula: Profitable ~ Epsilon * round). The model included simulation run as a random effect (formula: ~1 | run). The model's total explanatory power is substantial (conditional R^2 = 0.36) and the part related to the fixed effects alone (marginal R^2) is of 0.10. The model's intercept, corresponding to Epsilon = 0.01 and round = 0, is at 0.60 (95% CI [0.57, 0.63], p < .001). Within this model:

- The effect of Epsilon [0.05] is statistically non-significant and positive (β = 0.008, *95% CI* [-0.03, 0.05], *p* = .653; std. β = 0.25, *95% CI* [0.21, 0.28])

- The effect of Epsilon [0.1] is statistically non-significant and positive (β = 0.04, *95% CI* [-0.003, 0.07], *p* = .073; std. β = 0.23, *95% CI* [0.19, 0.26])

- The effect of Epsilon [0.2] is statistically non-significant and positive (β = 0.03, *95% CI* [-0.009, 0.07], *p* = .139; std. β = 0.30, *95% CI* [0.26, 0.33])

- The effect of Epsilon [0.5] is statistically non-significant and negative (*beta* = -0.02, *95% CI* [-0.06, 0.02], *p* = .369; std. β = 0.23, *95% CI* [0.20, 0.27])

- The effect of round is statistically significant and positive (β = 0.009, *95% CI* [0.008, 0.01], *p* < .001; std. β = 0.08, *95% CI* [0.07, 0.09])

- The interaction effect of round on Epsilon [0.05] is statistically significant and positive (β = 0.02, *95% CI* [0.01, 0.02], *p* < .001; std. β = 0.13, *95% CI* [0.12, 0.14])

- The interaction effect of round on Epsilon [0.1] is statistically significant and positive (β = 0.01, *95% CI* [0.01, 0.01], *p* < .001; std. β = 0.11, *95% CI* [0.10, 0.12])

- The interaction effect of round on Epsilon [0.2] is statistically significant and positive (β = 0.02, *95% CI* [0.02, 0.02], $p < .001$; std. β = 0.15, *95% CI* [0.14, 0.16])

- The interaction effect of round on Epsilon [0.5] is statistically significant and positive (β = 0.02, *95% CI* [0.02, 0.02], $p < .001$; std. β = 0.14, *95% CI* [0.13, 0.15])

Standardized parameters were obtained by fitting the model on a standardized version of the dataset. 95% Confidence Intervals (CIs) and p-values were computed using a Wald z-distribution approximation.

1.1.1.4 Model predicting the number of agents on secure neutral option with round and epsilon parameter

We fitted a Poisson mixed model (estimated using ML and BOBYQA optimizer) to predict choice of the secure neutral option with Epsilon and round (formula: SecureNeutral ~ Epsilon * round). The model included run as a random effect (formula: ~1 | run). The model's total explanatory power is substantial (conditional $R^2 = 0.34$) and the part related to the fixed effects alone (marginal R^2) is of 0.05. The model's intercept, corresponding to Epsilon = 0.01 and round = 0, is at 0.40 (*95% CI* [0.37, 0.44], $p < .001$). Within this model:

- The effect of Epsilon [0.05] is statistically non-significant and positive (β = 0.008, *95% CI* [-0.04, 0.06], $p = .744$; std. β = -0.43, *95% CI* [-0.48, -0.38])

- The effect of Epsilon [0.1] is statistically non-significant and positive (β = 0.02, *95% CI* [-0.03, 0.08], $p = .348$; std. β = -0.17, *95% CI* [-0.22, -0.13])

- The effect of Epsilon [0.2] is statistically non-significant and positive (β = 0.04, *95% CI* [-0.009, 0.09], $p = .111$; std. β = -0.31, *95% CI* [-0.36, -0.26])

- The effect of Epsilon [0.5] is statistically non-significant and negative (β = -0.002, *95% CI* [-0.05, 0.05], $p = .935$; std. β = -0.42, *95% CI* [-0.46, -0.37])

- The effect of round is statistically significant and positive (β = 0.005, *95% CI* [0.004, 0.006], $p < .001$; std. β = 0.05, *95% CI* [0.04, 0.06])

- The interaction effect of round on Epsilon [0.05] is statistically significant and negative (β = -0.03, *95% CI* [-0.03, -0.03], $p < .001$; std. β = -0.25, *95% CI* [-0.26, -0.23])

- The interaction effect of round on Epsilon [0.1] is statistically significant and negative (β = -0.01, *95% CI* [-0.01, -0.01], $p < .001$; std. β = -0.11, *95% CI* [-0.12, -0.10])

- The interaction effect of round on Epsilon [0.2] is statistically significant and negative (β = -0.02, *95% CI* [-0.02, -0.02], $p < .001$; std. β = -0.20, *95% CI* [-0.21, -0.18])

- The interaction effect of round on Epsilon [0.5] is statistically significant and negative (β = -0.03, *95% CI* [-0.03, -0.03], $p < .001$; std. β = -0.23, *95% CI* [-0.25, -0.22])

Standardized parameters were obtained by fitting the model on a standardized version of the dataset. 95% Confidence Intervals (CIs) and p-values were computed using a Wald z-distribution approximation.

1.2 Power Simulation

A power simulation was conducted based on H2 of Study 2 and Study 3. The data was simulated for two conditions that differed in their increase in probability to choose the best reward field (learning rate). The simulation was conducted for three distinct differences in learning rates (difference of 0.5 (small), 0.75 (medium), and 1 % (large) difference) and different numbers of groups with six members each (6, 10, 16, 20, 24, 30, 36, and 40). It should be noted that the power analysis relied on the preregistered statistical analysis, a logistic mixed model predicting choice of profitable card stack with condition (independence vs. cohesion) and round, with round, participant id, and group as random effects. This analysis is conducted based on individual data and not aggregated group data.

For 24 groups (N = 144), power for all learning rate differences exceeded 80 % (small: 86.2%, medium: 99%, large: >99%). Based on previous findings (Ritter *et al.*, 2021), a medium or large effect seems to be reasonable so that 24 groups overall risked overpowering the study. For additional economic reasons, a group number of 16 groups over all (8 per condition; N = 96) was chosen. Power for medium (93%) and large effects (> 99%) were still large, while also being reasonable for small effects (68%).

1.3 Additional Sample Information

1.3.1 Study 2

The study was advertised on online bulletin boards (i.e., bulletin board of the University of Goettingen, Facebook, and WhatsApp groups for psychology students in Geoettingen) and a blog that is read mostly by psychology students of the University of Goettingen. Most participants (*n* = 95) were university students. Participants were recruited from a participant data bank using ORSEE (Greiner, 2015). Subjects could participate in the study if (a) they were 18 years old or older, (b) gave written consent prior to participation, (c) had not participated in a similar study before, and (d) could participate in the study with their own laptop or PC and ensure a quiet environment during participation. Participants were reimbursed with a minimum of 2 Euro (show-up reward) and a maximum of 8 Euro. The reward was dependent on their behavior in the game. The earned rewards were transferred to participants' bank accounts after data collection was complete. If eligible, participants could also receive up to 1 participation credit.

1.3.2 Study 3

Most participants (n = 78) were university students that were recruited from a participant data base using ORSEE (Greiner, 2015) and via online advertisements on a virtual bulletin board and blog for psychology students of the University of Goettingen. Subjects could participate in the study if (a) they were 18 years old or older, (b) gave written consent prior to participation, (c) had not participated in a similar study before, and (d) could participate in the study with their own laptop or PC and ensure a quiet environment during participation. All participants were compensated with a base compensation of two euros if they appeared on time. They could then earn an additional bonus depending on their game performance. The earned rewards were transferred to participants' bank accounts after data collection was complete. If eligible, participants could also receive up to 1 participation credit.

1.4 R packages

Bates, D. *et al.* (2015) 'Fitting Linear Mixed-Effects Models Using lme4', *Journal of Statistical Software*, 67(1), pp. 1–48. Available at: https://doi.org/10.18637/jss.v067.i01.

Dahl, D.B. *et al.* (2019) *xtable: Export Tables to LaTeX or HTML*. Available at: https://CRAN.R-project.org/package=xtable.

Fox, J. and Weisberg, S. (2019) *An R Companion to Applied Regression*. Third. Thousand Oaks CA: Sage. Available at: https://socialsciences.mcmaster.ca/jfox/Books/Companion/.

Hervé, M. (2021) *RVAideMemoire: Testing and Plotting Procedures for Biostatistics*. Available at: https://CRAN.R-project.org/package=RVAideMemoire.

Jr, F.E.H. (2022) *Hmisc: Harrell Miscellaneous*. Available at: https://CRAN.R-project.org/package=Hmisc.

Kuznetsova, A., Brockhoff, P.B. and Christensen, R.H.B. (2017) 'lmerTest Package: Tests in Linear Mixed Effects Models', *Journal of Statistical Software*, 82(13), pp. 1–26. Available at: https://doi.org/10.18637/jss.v082.i13.

Lüdecke, D. (2020) *sjPlot: Data Visualization for Statistics in Social Science*. Available at: https://CRAN.R-project.org/package=sjPlot.

Lüdecke, D., Ben-Shachar, M.S., *et al.* (2021) 'performance: An R Package for Assessment, Comparison and Testing of Statistical Models', *Journal of Open Source Software*, 6(60), p. 3139. Available at: https://doi.org/10.21105/joss.03139.

Lüdecke, D., Patil, I., *et al.* (2021) 'see: An R Package for Visualizing Statistical Models', *Journal of Open Source Software*, 6(64), p. 3393. Available at: https://doi.org/10.21105/joss.03393.

Makowski, D. *et al.* (2020) 'Automated reporting as a practical tool to improve reproducibility and methodological best practices adoption', *CRAN* [Preprint]. Available at: https://github.com/easystats/report.

Makowski, D. *et al.* (2021) 'datawizard: Easy Data Wrangling', *CRAN* [Preprint]. Available at: https://easystats.github.io/datawizard/.

Neuwirth, E. (2014) *RColorBrewer: ColorBrewer Palettes*. Available at: https://CRAN.R-project.org/package=RColorBrewer.

Revelle, W. (2022) *psych: Procedures for Psychological, Psychometric, and Personality Research*. Evanston, Illinois: Northwestern University. Available at: https://CRAN.R-project.org/package=psych.

Tingley, D. *et al.* (2014) 'mediation: R Package for Causal Mediation Analysis', *Journal of Statistical Software*, 59(5), pp. 1–38. Available at: http://www.jstatsoft.org/v59/i05/.

Wei, T. and Simko, V. (2021) *R package 'corrplot': Visualization of a Correlation Matrix*. Available at: https://github.com/taiyun/corrplot.

Wickham, H. (2007) ‘Reshaping Data with the reshape Package’, *Journal of Statistical Software*, 21(12), pp. 1–20. Available at: http://www.jstatsoft.org/v21/i12/.

Wickham, H. (2011) ‘The Split-Apply-Combine Strategy for Data Analysis’, *Journal of Statistical Software*, 40(1), pp. 1–29. Available at: http://www.jstatsoft.org/v40/i01/.

Wickham, H. (2016) *ggplot2: Elegant Graphics for Data Analysis*. Springer-Verlag New York. Available at: https://ggplot2.tidyverse.org.

Wickham, H. *et al.* (2020) *dplyr: A Grammar of Data Manipulation*. Available at: https://CRAN.R-project.org/package=dplyr.

Wickham, H. and Bryan, J. (2022) *readxl: Read Excel Files*. Available at: https://CRAN.R-project.org/package=readxl.

Wilke, C.O. (2020) *cowplot: Streamlined Plot Theme and Plot Annotations for ‘ggplot2’*. Available at: https://CRAN.R-project.org/package=cowplot.

1.5 Additional information on questionnaire measures

Self-confidence was measured using the Multidimensionale Selbstwertskala (Schütz, Rentzsch and Sellin, 2006), a multidimensional self-description scale for measuring facets of self-esteem. The scale contains six subscales of which four were included in this study: emotional self-esteem (ESWS) as general self-esteem, social self-esteem (SWKO and SWKR), and performance-related self-esteem (LSWS). Self-esteem of physical attractiveness (SWPA) and the self-esteem athleticism (SWSP) were not included in this study as they are not relevant to the experimental context.

Decisiveness was measured using the Decisiveness Scale, a part of the Need for Closure Scale by Webster and Kruglanski (1994), adapted by Roets and Van Hiel (2007). The scale was translated to German by the authors and translation quality was checked through independent back-translation.

Achievement motivation was measured using the short version of the Leistungsmotivationsinventar (LMI-K; Schuler and Prochaska, 2001).

Risk propensity was measured with the R-1 measure by Beierlein et al. (2014), a one item scale.

2 Supplementary Figures

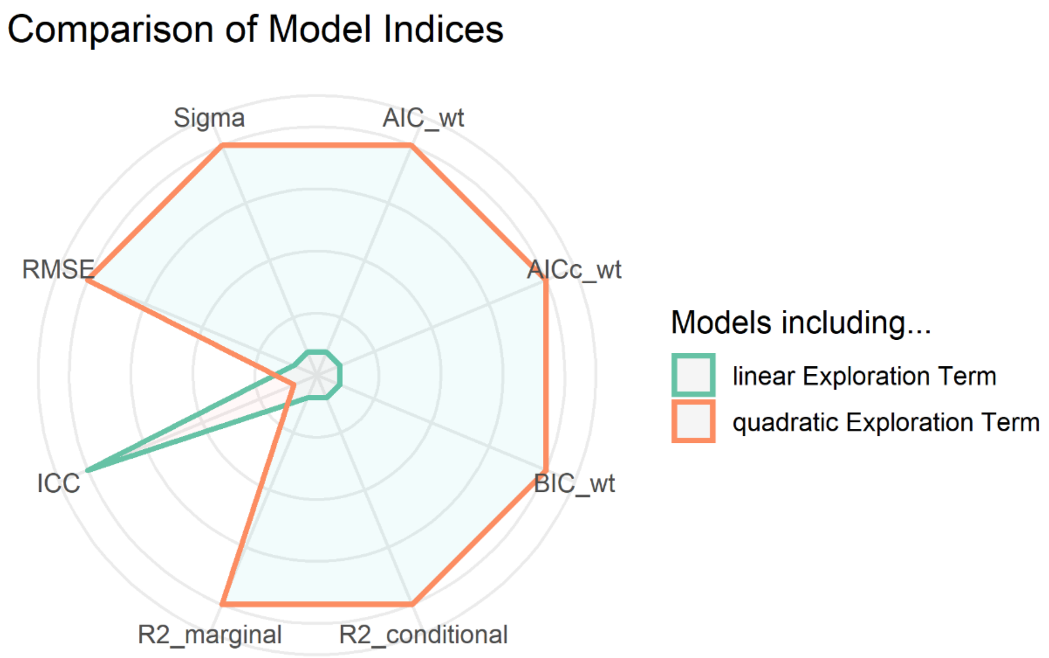

Supplementary Figure 1. Weights of two models of the relationship of exploration and decision quality (H5) on eight different model indices are plotted: AIC, corrected AIC, BIC, conditional R2, marginal R2, intra-class correlation (ICC), root-mean-square error (RMSE), and residual standard deviation (Sigma).

3 References for Supplementary Material

Beierlein, C. *et al.* (2014) 'Eine Single-Item-Skala zur Erfassung von Risikobereitschaft: Die Kurzskala Risikobereitschaft-1 (R-1)', *GESIS-Working Papers*, 2014/34.

Greiner, B. (2015) 'Subject pool recruitment procedures: organizing experiments with ORSEE', *Journal of the Economic Science Association*, 1(1), pp. 114–125. Available at: https://doi.org/10.1007/s40881-015-0004-4.

Ritter, M. *et al.* (2021) 'How collective reward structure impedes group decision making: An experimental study using the HoneyComb paradigm', *PLOS ONE*. Edited by T.A. Han, 16(11), p. e0259963. Available at: https://doi.org/10.1371/journal.pone.0259963.

Roets, A. and Van Hiel, A. (2007) 'Separating Ability From Need: Clarifying the Dimensional Structure of the Need for Closure Scale', *Personality and Social Psychology Bulletin*, 33(2), pp. 266–280. Available at: https://doi.org/10.1177/0146167206294744.

Schuler, H. and Prochaska, M. (2001) *Leistungsmotivationsinventar: Dimensionen berufsbezogener Leistungsorientierung*. Hogrefe.

Schütz, A., Rentzsch, K. and Sellin, I. (2006) 'Multidimensionale Selbstwertskala: MSWS; Manual'.

Webster, D.M. and Kruglanski, A.W. (1994) 'Individual Differences in Need for Cognitive Closure', p. 14.
